# A draft map of the mouse pluripotent stem cell spatial proteome

**DOI:** 10.1038/ncomms9992

**Published:** 2016-01-12

**Authors:** Andy Christoforou, Claire M. Mulvey, Lisa M. Breckels, Aikaterini Geladaki, Tracey Hurrell, Penelope C. Hayward, Thomas Naake, Laurent Gatto, Rosa Viner, Alfonso Martinez Arias, Kathryn S. Lilley

**Affiliations:** 1 Department of Biochemistry, Cambridge Centre for Proteomics, University of Cambridge, Tennis Court Road, Cambridge CB2 1QR, UK; 2 Department of Genetics, University of Cambridge, Downing Street, Cambridge CB2 3EH, UK; 3 Department of Biochemistry, Computational Proteomics Unit, University of Cambridge, Tennis Court Road, Cambridge CB2 1QR, UK.; 4 Department of Pharmacology, University of Pretoria, Arcadia 0007, Republic of South Africa; 5 Thermo Fisher Scientific, 355 River Oaks Pkwy, San Jose, California 95314, USA

## Abstract

Knowledge of the subcellular distribution of proteins is vital for understanding cellular mechanisms. Capturing the subcellular proteome in a single experiment has proven challenging, with studies focusing on specific compartments or assigning proteins to subcellular niches with low resolution and/or accuracy. Here we introduce hyperLOPIT, a method that couples extensive fractionation, quantitative high-resolution accurate mass spectrometry with multivariate data analysis. We apply hyperLOPIT to a pluripotent stem cell population whose subcellular proteome has not been extensively studied. We provide localization data on over 5,000 proteins with unprecedented spatial resolution to reveal the organization of organelles, sub-organellar compartments, protein complexes, functional networks and steady-state dynamics of proteins and unexpected subcellular locations. The method paves the way for characterizing the impact of post-transcriptional and post-translational modification on protein location and studies involving proteome-level locational changes on cellular perturbation. An interactive open-source resource is presented that enables exploration of these data.

## Abstract (web summary)

The spatial location of proteins within a cell is a key element of protein function. Here the authors describe hyperLOPIT—a proteomics workflow that allows the simultaneous assignment of thousands of proteins to subcellular niches with high resolution—and apply it to mouse pluripotent stem cells.

## Graphical abstract

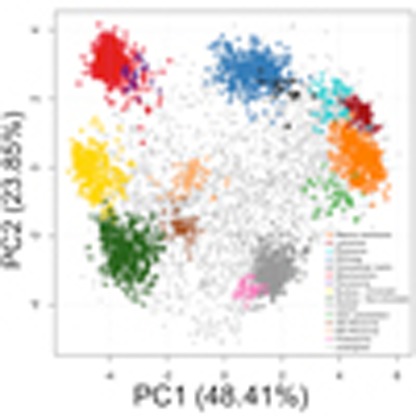

Pluripotent mouse embryonic stem (ES) cells are self-renewing clonal populations derived from blastocysts, which can be differentiated into the ensemble of cell types of the organism^1^,^2^. Their study is central to developmental biology and the emerging field of regenerative medicine. Currently our understanding of the biology of ES cells is deeply rooted in our knowledge of their transcriptomes, epigenetics and underlying gene regulatory networks, which have created a foundation for understanding pluripotency and the transition to differentiation^3^,^4^.

There is evidence that post-transcriptional events such as signalling, adhesion, protein turnover and post-translational modification make a significant contribution to the regulation of differentiation^5^,^6^,^7^,^8^, yet their precise roles in this process, and how they interact with each other and with the transcriptional machinery, remain open questions. The transition from self-renewal to differentiation is also associated with major changes in cell morphology, and therefore some of the effects of these post-transcriptional processes must be associated with changes in intracellular organization. Understanding the subcellular distribution of proteins and other biomolecules, and how the distribution changes with cell state, is therefore of paramount importance for the delineation of post-transcriptional processes in ES cells.

Protein localization is typically determined by immunocytochemistry or by monitoring fluorescent fusion proteins by confocal microscopy. While these approaches are valuable and well-established, there are certain limitations to their applicability. Immunocytochemistry is dependent on the availability of high-specificity and high-sensitivity antibodies, while fluorescent fusion proteins are vulnerable to aberrant localization due to the effect of the fusion moiety on protein topology^9^,^10^. These limitations can be overcome with complementary technologies such as protein mass spectrometry (MS), which offers the capability to assay thousands of proteins simultaneously and in their native state^11^.

Localization of organelle proteins by isotope tagging (LOPIT) is a quantitative proteomics method for the high throughput and simultaneous characterization of multiple subcellular compartments, without the requirement for total purification of compartments of interest^12^. LOPIT combines biochemical fractionation by density-gradient ultracentrifugation, sample multiplexing by *in vitro* covalent labelling, and liquid chromatography-mass spectrometry. In LOPIT, cells are first lysed under detergent-free conditions so that there is minimal disruption to organelle integrity. Membranes are then separated based on their characteristic buoyant densities by ultracentrifugation. Although organelles do not partition into discrete purified fractions, different organelles display distinct enrichment patterns. Fractions representing peak enrichment for organelles of interest are selected for proteolytic digestion. The resulting peptides are differentially labelled with amine-reactive tandem mass tag (TMT) reagents^13^, which allow peptides derived from each fraction to be distinguished by mass spectrometry. The relative abundance of a peptide can be determined by its TMT reporter ion profile, which recapitulates the distribution of the protein across the fractionation scheme. Proteins residing in the same subcellular niche would be expected to co-distribute, and therefore present similar TMT reporter ion profiles^14^. Classification algorithms are then used to assign proteins to subcellular compartments based on correlation with organellar marker proteins.

Here we significantly extend the LOPIT concept with novel approaches for sample preparation, mass spectrometry data acquisition and multivariate analysis. This new workflow, named hyperplexed LOPIT (hyperLOPIT), benefits from several recent technological advancements. First, the development of neutron-encoded isotopologue variants of TMT has increased the multiplexing capacity of isobaric tagging experiments to 10 samples^15^. These additional labels have enabled more subcellular fractions to be sampled, allowing for a more elaborate fractionation scheme that reaches sub-organellar levels of resolution. Second, quantitative accuracy of TMT-based applications is significantly improved by mass spectrometry data acquisition using synchronous precursor selection MS^3^ (SPS-MS^3^; see Supplementary Fig. 1 for a detailed overview of this method). Multivariate approaches such as hyperLOPIT represent particularly demanding applications of TMT quantification, as consistently high accuracy and precision are necessary for co-localized proteins to display correlated TMT distributions^16^. We have therefore incorporated SPS-MS^3^ acquisition on the Orbitrap Fusion Tribrid mass spectrometer (Thermo Fisher Scientific) into the hyperLOPIT pipeline, and demonstrate that it greatly improves spatial resolution and the reliability with which protein localization may be determined. Finally, we have extended our data analysis platform to facilitate rapid interrogation of the data by providing an easy-to-use graphical user interface.

We apply the hyperLOPIT workflow to a population of self-renewing mouse ES cells. The result is comprehensive coverage of the subcellular proteome with unprecedented spatial resolution, enabling the mapping of components of organelles, transitory proteins, multi-protein complexes, signalling pathways and families of functionally related proteins. The presented data offer hitherto unknown subcellular detail about a population of self-renewing stem cells. We observe the dynamic nature of Golgi apparatus proteins, noting sets of Golgi marker proteins that are distributed amongst other subcellular structures, an observation we support by microscopy. We also demonstrate that additional insights into published interactomes and focused protein-localization studies can be attained by integrative analysis with LOPIT. HyperLOPIT offers a spatial scaffold onto which other high content proteomics data sets can be mapped yielding added value to complementary data. We give an example of this functionality by combining the data presented here with a recent data set of surface proteins captured using chemical tagging.

## Results

### Biochemical fractionation of mouse ES cells

**Figure 1:**
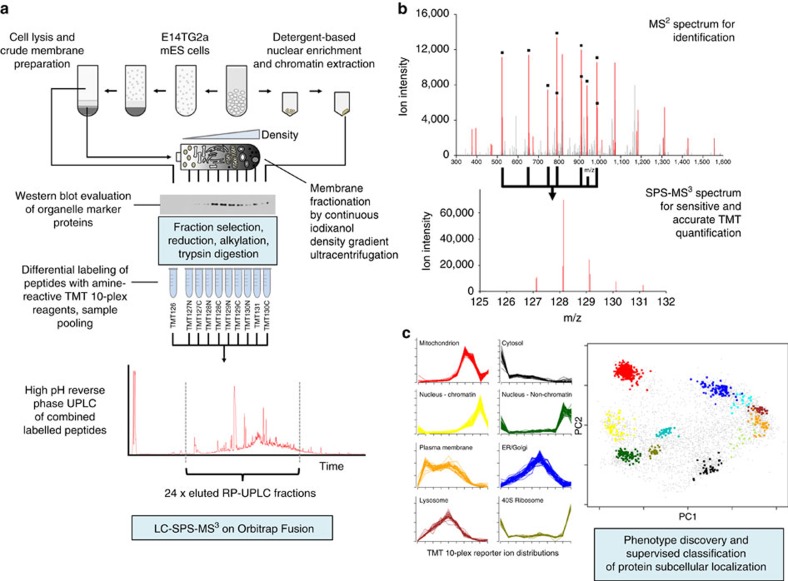
Schematic of the hyperLOPIT workflow. (**a**) Ten enriched subcellular fractions were generated from E14TG2a murine ES (mES) cells. Peptides derived from each fraction were differentially labelled with TMT 10-plex reagents, and analysed by two-dimensional LC-SPS-MS^3^. (**b**) SPS-MS^3^ boosts the sensitivity of MS^3^-based TMT quantification, while preserving the gains in quantitative performance relative to conventional MS^2^, by selecting multiple peptide fragments rather than a single ion for MS^3^ analysis. (**c**) TMT reporter ion distributions recapitulate the distribution of proteins across the fractionation scheme. Different organelles display characteristic distributions that may be used to determine their residents. The high dimensional data are presented in two-dimensions by PCA to provide an intuitive visualization of organelle separation.

An overview of the hyperLOPIT workflow is shown in Fig. 1. To create a method fit for the purpose of capturing cell-wide proteome localization data, we first improved the LOPIT workflow as follows: to increase subcellular resolution, extensive subcellular fractionation of pluripotent E14TG2a cells was performed. First, crude membranes were separated from the soluble fraction, enriched in cytosolic proteins. Crude membranes were then separated into organelle-enriched fractions by equilibrium density gradient centrifugation, within which organelles adopt specific distribution profiles consistent with their respective buoyant densities. A portion of the cell culture was also used to enrich chromatin-associated proteins with a parallel workflow based on detergent permeabilization^16^. Ten fractions were chosen to best represent peak organelle densities, labelled with TMT reagents and processed as described in the Methods section. We acquired three biological replicates of hyperLOPIT data, each with a slightly different selection of subcellular fractions for TMT 10-plex labelling. The first replicate placed greater emphasis on resolving low density-gradient fractions that are enriched in secretory pathway components, while the second and third replicates placed greater emphasis on separation of the denser organelles such as the mitochondrion and peroxisome.

### SPS-MS^3^ enhances quantitative performance

TMT quantification by conventional tandem mass spectrometry (MS^2^) suffers from impaired quantitative accuracy and precision due to interference from contaminant peptides with similar chromatographic and mass-to-charge properties to the target peptide. Improved quantitative performance is achieved with an additional round of ion selection and fragmentation to purify the analyte from which TMT quantification is derived^17^,^18^, but such MS^3^-based methods result in substantially reduced sensitivity^19^. SPS-MS^3^ balances the quantitative gains of MS^3^ quantification with the sensitivity required for proteome-wide analysis^20^,^21^. Whereas in conventional MS^3^ a single peptide fragment ion is selected for quantification, by using isolation waveforms with multiple frequency ‘notches' to collect multiple peptide fragments, SPS-MS^3^ improves ion statistics for quantification (Supplementary Fig. 1).

To assess the impact of SPS-MS^3^ on quantitative performance, we evaluated the effect of the number of SPS-MS^3^ ‘notches' by comparing quantification derived from conventional MS^2^, conventional MS^3^ and SPS-MS^3^ data acquisition. Adjusting the number of SPS-MS^3^ notches altered the balance between quantitative performance and sensitivity. Increasing the number of notches augmented the TMT reporter ion signal intensity, and therefore the proportion of quantifiable spectra, but also reintroduces contaminant ions that distort quantification. Fewer notches result in lower reporter ion signal, with concomitant reduction in the number of quantifiable spectra. We found SPS-MS^3^ with 10 precursors `notches' to represent a suitable balance for global analysis, with 92.8% of the acquired SPS-MS^3^ spectra yielding TMT reporter ion counts >1 × 10^5^—a comparable figure to that obtained with conventional MS^2^ acquisition (Supplementary Table 1).

**Figure 2:**
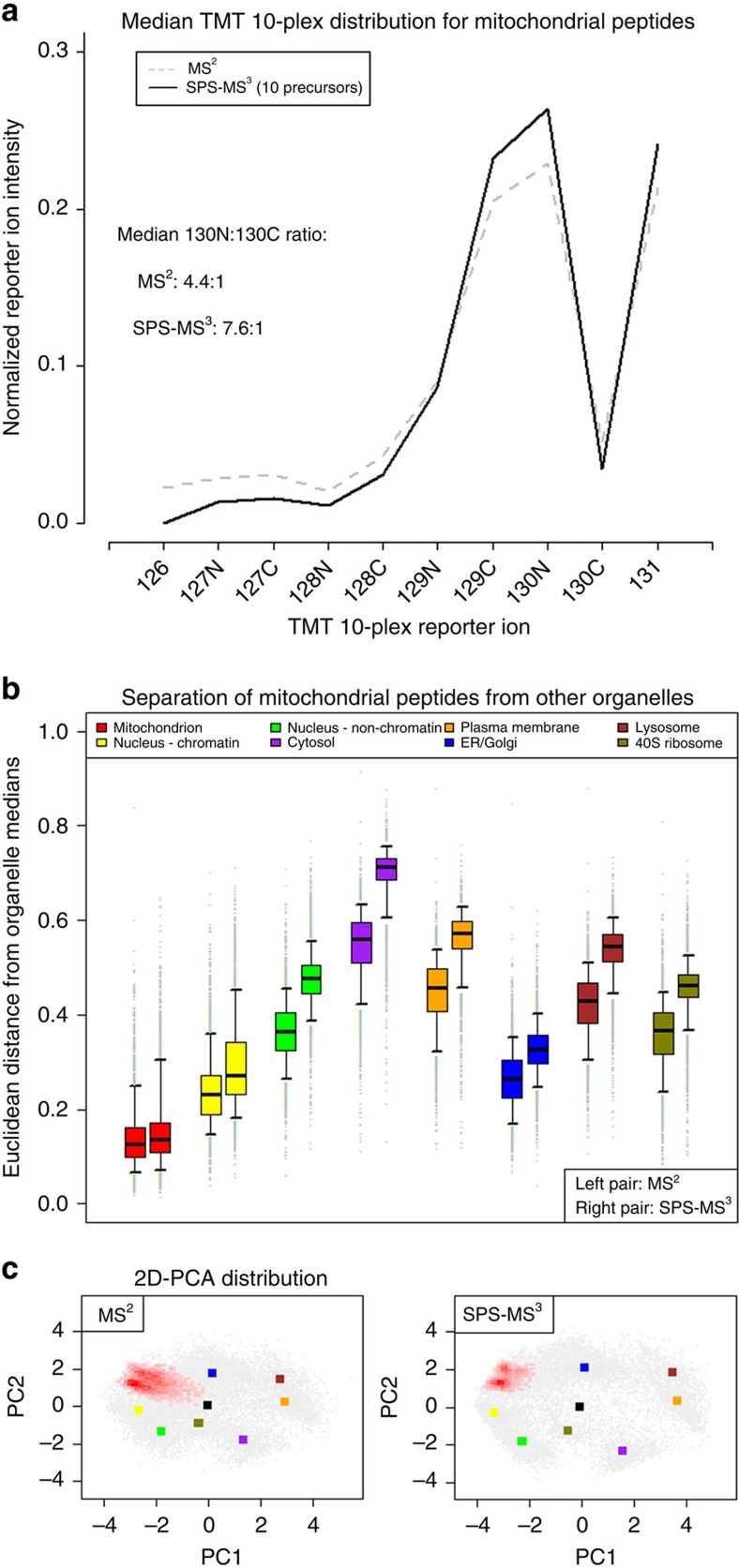
SPS-MS^3^ enhances resolution of the mitochondrion from other organelles. (**a**) SPS-MS^3^ results in higher quantitative accuracy. The ratio between TMT channels expected to be enriched in mitochondria (129C and 130N) and channels that are expected to be depleted (for example, 130C) is markedly increased by SPS-MS^3^. (**b**) The distance of mitochondrial PSMs from the median of eight organellar phenotypes. The distance of mitochondrial PSMs from the median mitochondrial distribution does not change significantly between MS^2^ and SPS-MS^3^, but the distances from other organelles are all significantly increased in SPS-MS^3^ (Wilcoxon rank sum test *P* value <2.2 × 10^−16^), indicating greater organellar resolution. (**c**) When represented in two dimensions by PCA, the mitochondrial PSMs (red heat map) show less skew towards the origin, due to the improved specificity of SPS-MS^3^ quantification. The median positions of other organelles are represented by coloured squares and individual proteins by grey points. Similar plots demonstrating the enhanced resolution of other organelles are presented in Supplementary Fig. 2.

We then compared E14TG2a hyperLOPIT data sets acquired with conventional MS^2^, and SPS-MS^3^ with 10 notches. When comparing peptides derived from proteins with well curated localization, we observed SPS-MS^3^ acquisition resulted in a significant improvement in quantitative accuracy over conventional MS^2^ acquisition (Fig. 2 and Supplementary Figs 2 and 3). This gain in quantitative performance resulted in greater resolution of organelles from one another, and from the median of the total peptide population; tendency towards a unified distribution being characteristic of distorted TMT quantification (Supplementary Fig.18)^16^,^22^.

While the increase in instrument duty cycle to perform SPS-MS^3^ decreased the number of quantified peptide-spectrum matches (PSMs) from 137,912 to 61,090, the difference between the number of quantifiable protein groups was less substantial (7,114 versus 5,489)—a tolerable compromise given the gains in spatial resolution (Supplementary Fig.17).

### HyperLOPIT provides an overview of protein localization

Over 6,000 protein groups were quantified in each of the three replicate experiments (Supplementary Data 1). The fractionation patterns observed in experiments 1 and 2 are highly consistent, while the resolution of secretory pathway organelles in experiment 3 was reduced owing to lower protein yields (Supplementary Fig. 4). Despite the lower resolution of some compartments in experiment 3, classification of proteins to subcellular compartments was highly reproducible, with <5% of proteins assigned contradictory localizations across the three experiments (Supplementary Fig. 5).

The intersect of experiments 1 and 2 (5,032 protein groups, Supplementary Data 1) was treated as a 20-plex data set for the analysis discussed in this article, which has previously been demonstrated to improve the attainable spatial resolution^23^,^24^. Experiment 3 was not included, as little additional resolution was obtained by further data fusion.

**Figure 3:**
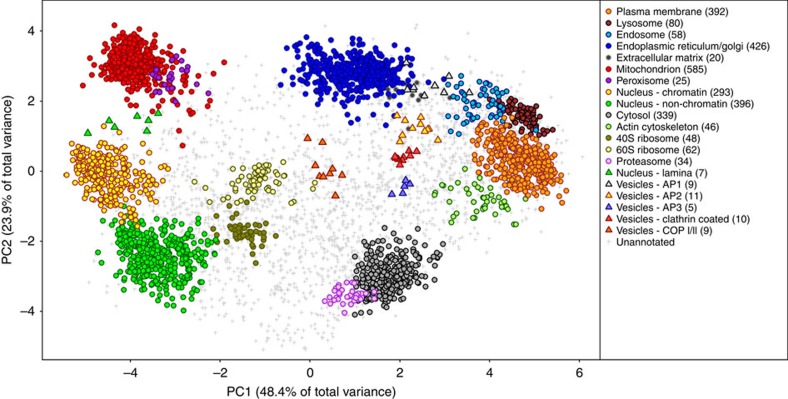
PCA representation of E14TG2a mouse ES cell hyperLOPIT data. Each data point represents a protein group, and proteins with correlated TMT reporter ion distributions cluster together in PCA space. Coloured circles represent subcellular compartments that have been classified by SVM and coloured triangles represent smaller organellar phenotypes that were manually curated. While two dimensions describe much of the spatial resolution in these data, separation of some compartments, such as the mitochondrion and peroxisome, is only apparent in the lower principal components (Supplementary Fig. 5).

Using the pRoloc data analysis pipeline^25^, an initial application of novelty detection^26^ was conducted to identify and confirm the presence of organelle clusters in an unbiased data-specific manner, followed by supervised classification using a support vector machine (SVM) for final protein-organelle assignment. Applying SVM scoring thresholds based on concordance with gene ontology annotation, the steady-state localization of 2,855 out of a total of 5,032 protein groups were unambiguously determined (Fig. 3 and Supplementary Figs 6 and 7). Sub-nuclear resolution was also obtained, with proteins localized to chromatin, non-chromatin nuclear and nuclear lamina displaying distinct quantitative distributions. Manual curation of the data set also revealed co-localization of proteins for several other subcellular niches, including coatamer and clathrin-coated vesicles, and cytoskeletal fragments (Fig. 3).

Given the breadth of proteome coverage and subcellular resolution, hyperLOPIT can be used to investigate the organization of cells at multiple levels of scope. Insights into cell behaviour may be drawn from the localization of individual proteins, protein families and even functional networks, or by evaluating the protein content of particular subcellular compartments. In the following sections, we discuss how some of the layers of data can be interpreted, and the insights into the organization of pluripotent ES cells that may be drawn.

### Organelle membership

The hyperLOPIT workflow enabled unambiguous assignment of 2,855 proteins to 14 discrete organellar and sub-organellar compartments in a single experiment. This number amounted to over 50% of the proteins identified, with the remainder displaying intermediate distributions as described in the Proteins in transit section. The classified proteins represent both residents of an organelle, as well as transient traffickers or cargo proteins that are captured in this position under these experimental conditions and in this particular cell type. The catalogue of organelle members is therefore context specific and must be viewed as a subcellular snapshot of a dynamic system.

Approximately 83% of proteins classified by hyperLOPIT carry localization-specific gene ontology annotation, with only 39% of these proteins annotated based on direct assay evidence. This data set therefore provides new experimental evidence for the localization of 1,775 murine proteins, ∼350 of which currently lack organelle-specific localization information in the UniProt database (Supplementary Data 1). It should be noted that these values include proteins that are annotated as cytoplasmic. Since the term cytoplasm incorporates the cytosol, cytoskeleton, organelles and other cellular features other than the nucleus and cell surface, such annotation lacks the specificity to be suitably informative in this context. HyperLOPIT is therefore a useful tool for supplementing database annotation of protein localization with high throughput. Another key strength of the approach is that protein localization may be determined in particular cell type(s) and cell state(s) of interest—information that is typically lacking in gene ontology annotation or computational prediction of localization.

We compared the protein localizations determined by hyperLOPIT with another recently published plasma membrane proteome for the same cell line. Bausch-Fluck *et al.*^27^ identified plasma membrane proteins by cell surface biotinylation and affinity purification-mass spectrometry. In this study the authors categorized their cell capture data into three groups: High confidence, for proteins with UniProt keywords including ‘Cell junction', ‘Cell membrane' and ‘Secreted'; putative, for proteins with predicted transmembrane domains, but none of the above keywords assigned; and non-specific, for other identified proteins, which were assumed to be abundant contaminant proteins. Proteins labelled as high confidence in this study corroborate with their localization in the hyperLOPIT data, as almost all such proteins localize to the plasma membrane or endosomes—suggesting extensive recycling of some of these surface proteins. HyperLOPIT also confirms plasma membrane localization for many proteins labelled as putative in the Bausch-Fluck study (Supplementary Fig. 8). The third category, non-specific proteins, consists mostly of proteins found in non-surface localizations by hyperLOPIT. The strong correlation of results demonstrates that hyperLOPIT may also be used as an orthogonal validation method for targeted localization studies.

### Proteins in transit

Not all proteins are expected to partition into discrete subcellular compartments. While over half of the quantified proteins were classifiable to a single and unambiguous location, many proteins were found to have distributions that did not closely correlate with those of the 14 classified subcellular compartments. These less discrete distribution patterns occur for several reasons.

First, proteins present in multiple compartments will adopt quantitative distributions that reflect their steady-state subcellular enrichment. β-catenin for example, has been robustly classified to the plasma membrane despite the fact that this protein is known to localize to the adherens junctions, cytosol and nucleus. Classification to the plasma membrane reflects its relative enrichment at the adherens junction in ES cells cultured under self-renewing conditions, which has been previously demonstrated by immunofluorescence microscopy^28^.

There are cases where the steady-state localization is not so skewed towards one of multiple compartments. For example, many components of the nuclear import and export machinery display distributions consistent with mixed localization between the cytosol and nucleus (Supplementary Fig. 9). These proteins were not classified as unambiguous residents of either the nucleus or cytosol; their distribution patterns accurately reflect their true subcellular localization. Other proteins displaying mixed localizations include signalling cascade effectors, such as Erk-2 and the adaptor protein Grb-2, components of the MAP kinase signalling pathway that distribute between the plasma membrane and cytosol, reflecting the dynamic transitions between the two locations. For some proteins with mixed localization the distribution patterns are quite complex. Mcl-1, a Bcl-2 family member protein, displays a three-way mixed localization between the endoplasmic reticulum, mitochondrion and nucleus, and therefore falls between the three organelle clusters in principal component analysis (PCA) space; this broad distribution is consistent with previous confocal microscopy analysis of its human orthologue^29^.

The steady-state distribution of transitory proteins can provide information about the state of the cell population. For example, Tfe3, a helix–loop–helix family transcription factor and modulator of the exit of ES cells from pluripotency, was observed with mixed localization between the cytoplasm and nucleus (Supplementary Fig. 9), consistent with immunocytochemistry data in ES cells. In the pluripotent state, Tfe3 localizes to both the nucleus and cytosol, and regulates the expression of key pluripotency factor Esrrb. In the early stages of differentiation, it is excluded from the nucleus^30^.

Second, intermediate subcellular distributions may represent proteins residing in organelles other than those used in the SVM classification. These compartments typically have their own physicochemical properties, and their resident proteins therefore co-localize, but are not sufficiently enriched for their distribution patterns to be fully resolved from those of other subcellular compartments. For example, known components of the clathrin-AP3 trafficking vesicles were found to co-localize away from other organelles and vesicles, but were not distinct from other proteins with mixed localization. Some proteins with correlated distributions to the clathrin-AP3 vesicles are plausible vesicular components, such as syntaxin-18, whereas others are transitory cytosolic and cytoskeletal proteins such as serine/threonine kinase Pak4.

Finally, the observed localization patterns may represent proteins that comprise or are tethered to the cytoskeleton. The fractionation pattern of the cytoskeleton is not easily predicted given its broad interconnectivity with other subcellular components. We observed groups of cytoskeletal proteins with a variety of distinct distributions, including actin, actin-capping modulators, microtubules, microtubule organizing centre components, dyneins, kinesins and myosins (Supplementary Fig. 10). While we did not perform classification on these smaller phenotypes, it is possible that uncharacterized proteins that co-localize with these niches are cytoskeletal components.

### Organelle structure

**Figure 4:**
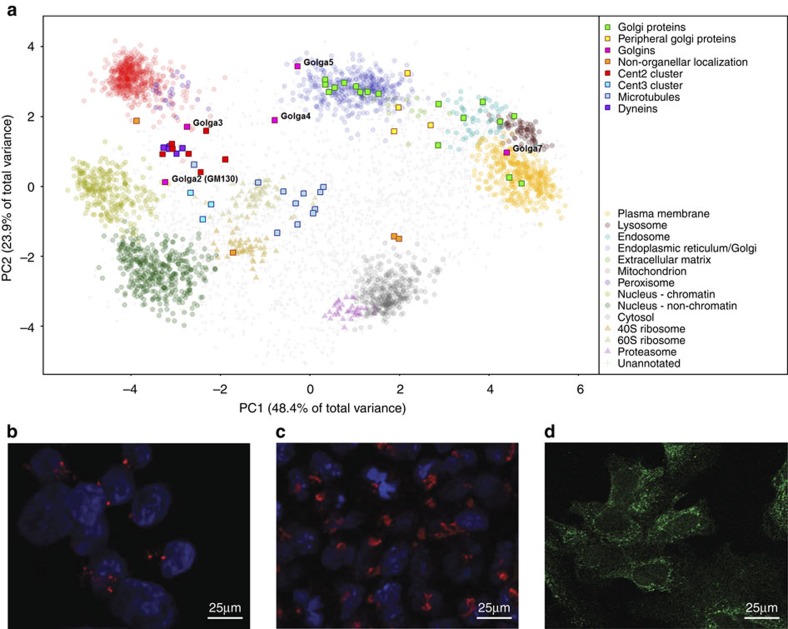
Steady-state location of Golgi apparatus marker proteins. (**a**) PCA plot of data with Golgi marker proteins overlaid Golgi proteins (green) peripheral Golgi proteins (yellow), Golgins (pink), microtubular proteins (light blue), dyneins (purple), centrin 2 and associated proteins (red) centrin 3 and associated proteins (mid-blue), Golgi proteins with aberrant localization (orange). (**b**) Immunocytochemistry using anti-GM130 (red) on a self-renewing population of E14TG2a cultured in serum and LIF showing a punctate localization not consistent with the Golgi Apparatus. (**c**) Immunocytochemistry using anti-GM130 on a population of E14TG2a after 4 days in N2B27, a media promoting neural differentiation. (**d**) Immunocytochemistry on a self-renewing population of E14TG2a cultured in serum and LIF using anti-KDEL (green; ER lumenal retention signal) to verify typical endoplasmic reticular structures encapsulating the nucleus.

The ability of hyperLOPIT to gain insight into very dynamic processes within the cell is exemplified by the Golgi apparatus. The position of well documented Golgi apparatus marker proteins reveals an unexpected observation about these cells. As can be seen from Fig. 4a, these markers fall into four categories; proteins that co-cluster with endoplasmic reticulum (ER) markers, proteins that lie between the ER/Golgi and endosomal compartments, proteins that cluster with plasma membrane markers, and proteins whose steady-state location does not correlate with any of the 14 classified compartments. The proteins that cluster along with ER markers are generally annotated as being *cis*-Golgi proteins, including alpha-mannosidase 2 (MA2A1)^31^ and members of the SNAP receptor complex (GOSR2)^32^. Proteins that cluster towards the endosomal markers are associated with the *trans*-Golgi, such as the copper-transporting ATPase (ATP7A)^33^ and vacuolar protein sorting-associated protein 45 (VSP45)^34^. Several proteins form part of the plasma membrane cluster including Ras-related protein Rab-6A (RAB6A)^35^ and Golgin 7 (GOGA7), which are thought to be involved in protein transport from the Golgi to the cell surface^36^. In general those proteins known to be peripherally associated with Golgi membranes lie in an intermediate position on the boundary of the ER/Golgi cluster, for example, the Golgi phosphoprotein GOLP3 and oxysterol-binding protein 1.

It is curious to note that some Golgi marker proteins occupy a steady-state position far removed from the ER/Golgi or endosomal vesicles. GM130 (GolginA2) is a *cis*-Golgi matrix protein, and is thought to be involved in regulation of centrosomes during interphase^37^. On the PCA plot (Fig. 4A), GM130 is intriguingly at a steady-state position between centriolar proteins such as pericentriolar material 1 protein (PCM1), centrin 2 (CETN2), spindle and centriole-associated protein (SPICE), pericentrin (PCNT) and another cluster of proteins containing gamma tubulin subunits and centrin 3 (CETN3)^38^,^39^,^40^. This is consistent with the role of GM130 to act as a tether between the Golgi and centrosomes in interphase^41^. To confirm our observations, we performed immunocytochemistry imaging with an anti-GM130 antibody, which supported the theory that this protein may be associated with the centriole in pluripotent mouse ES cells (mES; Fig. 4b). Interestingly, we noted that GM130 instead appeared to be associated with Golgi-like structures when the cells are transferred to a media promoting neural differentiation (N2B27 medium; Fig. 4c,d). The reasons behind the difference in distribution of GM130 observed between self-renewing and differentiating ES cells may reflect the relative dwell times of the two sets of cells in the stages of the cell cycle^42^, and it has previously been reported that mES cells have a truncated G1 phase^43^. The distribution of GM130 and is proximity to centriolar and pericentriolar matrix proteins may reflect that the majority of pluripotent cells are in the later stages of the cell cycle. Although the data do not show direct tethering of Golgi and centrosomal complex structures, this is just one example of how these data provide a snapshot of cellular behaviour.

### Protein complexes

**Figure 5:**
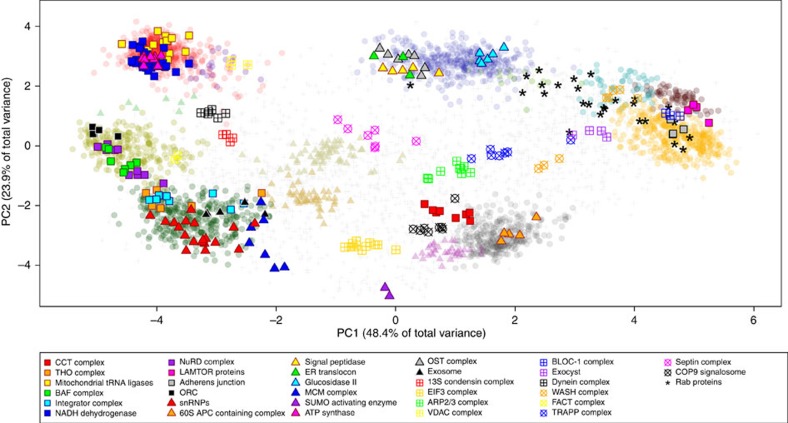
Examples of soluble and organellar protein complexes. Protein complexes are distributed throughout principal component space. Some soluble protein complexes display characteristic distributions that may reflect mixed localization, partitioning of the cytoskeleton by the fractionation scheme, or the unique sedimentation properties of these macromolecular structures. Sub-organellar distribution of complexes were observed in both ER, mitochondrion and nucleus.

In addition to organelles, we observed that many protein complexes display highly correlated distribution patterns. Co-localization of highly abundant macromolecular protein complexes, such as ribosomal subunits and the mitochondrial ATP synthase complex by LOPIT has been previously demonstrated^44^. The depth of proteome coverage in this study, coupled with the high subcellular resolution derived from precise quantification, enabled many more complexes to be detected. We selected 30 examples of highly curated protein complexes listed in KEGG^45^ and Reactome^46^,^47^, and annotated their distributions in the hyperLOPIT data (Fig. 5). There are many other protein complexes represented in the data set that we have not curated, but which readers of this study can explore according to their particular interest using pRolocGUI.

Proteins with multiple functional roles within the cell may be involved in more than one protein complex. Neither the extent of this, nor the distribution of components between these complexes is typically captured in large-scale complex purification studies. Inspection of the steady-state position of complex components within hyperLOPIT data indicates that components have differing positions from the core complex members, and may even indicate those that function in a regulatory manner. For example, the steady-state location of the TFIID complex is nuclear in these data, however, Taf7 has a steady-state distribution that is distinct from the other complex components (Supplementary Fig. 9). This may reflect the fact that Taf7 is thought to dissociate from the pre-initiation TFIID complex following initiation of transcription^48^. Another example is the exocyst complex, where Exoc8 is localized away from the core exocyst complex in these data, and co-distributes with its known binding partners Par6 and RalA^49^.

To demonstrate the utility of these data in evaluating other high-throughput data sets, we annotated the murine orthologues of proteins that were characterized in a census of human soluble protein complexes^50^. By adding this subcellular context to the analysis, we see that well-established components of protein complexes tend to co-localize, whereas novel assignments and putative uncharacterized complexes display more varied localization (Supplementary Fig. 11). Novel assignments that co-localize with the ‘core' complex might be assumed to be stable interactors, whereas those with different subcellular distributions may be transient interactors or false assignments. For example, two of eight novel components of the 39S mitochondrial ribosomal subunits assigned by Havugimana *et al.*^50^ were found to co-localize to the mitochondrion, whereas the remaining six were distributed in the nucleus, cytosol and secretory pathway (Supplementary Fig. 11C). The two co-localized proteins (Ict1 and Mrp63) both feature mitochondrial signal peptides and have molecular functions consistent with translation. The mouse and human variants of the six other putative interactors do not contain a distinct signal peptide, and are therefore probable false assignments in the census data set.

### Functional networks

The high proteome coverage generated by hyperLOPIT enabled us to determine the localization of many proteins associated with pluripotency and differentiation, including components of the core transcriptional network of pluripotent cells such as Sox2, Oct4 and Nanog. Also, impressively, the subcellular distributions of the components of FGF/MAPK, canonical Wnt, Notch, BMP/SMAD, Nodal, Ras and Hippo signalling pathways are apparent, paving the way for using hyperLOPIT to determine modulation in the location of signalling proteins and their effectors on activation/deactivation of multiple signalling pathways during, for example, differentiation (Supplementary Fig. 12). During self-renewal, Wnt/β-catenin and FGF/MAPK signalling are maintained at a low level^28^. Consistent with this, we observe that β-catenin is firmly located in the plasma membrane and that Sprouty proteins, negative regulators of FGF/ERK signalling, are also associated with the membrane, where they act to inhibit the early stages of FGF signalling. We also observe extranuclear localization of Smad2/5, consistent with the known low Nodal/Activin signalling in self-renewal^42^. To our knowledge this is the first instance in which it is possible to have a snapshot of all signalling pathways in one cell.

To evaluate the spatial distribution of interaction partners for the core pluripotency transcription factor triad of Oct4, Sox2 and Nanog, we overlaid information from three high-throughput protein–protein interaction studies onto the E14TG2a hyperLOPIT data set^51^,^52^,^53^. As might be expected, the seven interaction partners that were common to all three bait proteins displayed steady-state enrichment to chromatin (Supplementary Fig. 13D). Three of these common interaction partners (Chd4, Mta1 and Mta2) are components of the NuRD complex, demonstrating the importance of chromatin remodelling for maintenance and exit from the pluripotent state^54^. While the interactome was found to be chromatin centric, each of the transcription factors was also reported to interact with proteins that we observed with subcellular enrichment away from chromatin by hyperLOPIT (Supplementary Fig. 13A–C). The functional implications of these non-chromatin interactors are of potential interest, as their differential localization suggests that trafficking of the interaction partners into the nucleus, or of the transcription factor to extranuclear locations, plays a role in modulating the interaction. Alternatively, protein–protein interactions that are far removed may be indicative of a false discovery, as described previously for the 39S ribosomal subunit.

### Protein isoforms

HyperLOPIT can also provide information on the localization of protein isoforms. The impact of post-transcriptional modification on protein location has been previously documented^55^, however observation of the differences in subcellular location of closely related isoforms has not been previously possible using LOPIT, as the accuracy of quantification was insufficient to reliably characterize the few peptides, or often single peptide, which distinguish protein isoforms. With the enhanced quantitative performance of TMT quantification by SPS-MS^3^, measurements based on few peptides still yield reliable reporter ion distributions that allow us to characterize localization. We observed unique evidence for 25 and 26 pairs of protein isoforms (distinct proteins sharing the same gene name) in experiments 1 and 2, respectively (Supplementary Data 1). Some of the detected protein isoforms were found to co-localize, while others were found to be differentially localized.

For example, two murine isoforms of Leucine aminopeptidase 3 (Lap3) have been reported to arise from alternative translational initiation codons in the same mRNA; a ‘long' canonical isoform, and a ‘short' isoform with a 31 residue N-terminal truncation. We observed the ‘long' isoform of Lap3 with unambiguous mitochondrial localization, whereas the ‘short' isoform was found with a steady-state distribution between the cytosol and plasma membrane (Supplementary Fig. 14A). Protein-localization algorithms based on primary sequence, such as WoLF PSORT (ref. 56), support the observation that the long isoform of Lap3 localizes to mitochondria, whereas the short isoform does not (although the specific localization of the short isoform within the cytoplasm is not predictable from sequence alone). This suggests that the two isoforms, while sharing a common catalytic activity, fulfil separate biological roles due to differential localization. The differential localization is achieved through alternative translation that incorporates or excludes an N-terminal mitochondrial target signal. Further credence is given to the dual localization determined by hyperLOPIT by the fact that predicted functional partners of Lap3 (Anpep, Cat, Ggt1, Gss, Hspd1 and Pycr), as reported by the STRING protein interaction database (v9.1 (ref. 57)), are found to localize to the mitochondrion, plasma membrane, and cytosol—the three subcellular compartments described by the steady-state localization of the two Lap3 isoforms.

The differential localization of some other isoforms is not as straightforward to interpret. Two isoforms of Dnmt1, a DNA (cytosine-5)-methyltransferase that modulates ES cell pluripotency by maintaining CpG methylation patterns^58^ were identified; a canonical ‘long' isoform, and ‘short' isoform with an 118 residue N-terminal truncation that removes the Dmap interacting domain. The canonical isoform distribution was consistent with localization to chromatin, and closely co-localizes with Dmap1. The short isoform also appears to localize to the nucleus, but with an atypical distribution profile that while most similar to chromatin, is distinct from the typical chromatin profile (Supplementary Fig. 14B). The significance of this differential nuclear distribution is not immediately clear^59^.

While the number of isoforms pairs identified here is relatively modest, the depth of attainable proteome coverage will increase as the speed and sensitivity of high-resolution accurate mass spectrometry continues to advance, and will allow more of these isoforms to be detected. Accurate quantification of proteins based on a single peptide measurement will also permit characterization of post-translationally modified peptides. The ability to functionally characterize post-translational variants of proteins is an exciting avenue of research, as such variants introduce an additional dimension of biochemical complexity that is not easily evaluated with high throughput.

## Discussion

The hyperLOPIT technique is a systematic and accurate assay for characterizing the localization of thousands of proteins in a single experiment, and is applicable to many biological model systems. Application of this technology to a self-renewing population of ES cells has generated the most extensive analysis of protein localization in a stem cell line to date. The data contain information about organelle residency of proteins, sub-organellar structure, the impact of isoform status on location and dynamic localization of proteins. This creates a reference for mapping proteins relative to each other in pluripotency and differentiation.

HyperLOPIT data may also be used to provide a spatial context for pre-established protein complexes and functional networks. For example, our results provide molecular support for some functional observations about the state of several signalling pathways in ES cells. HyperLOPIT can also be used as an orthogonal approach for validation of targeted localization studies, as we have demonstrated with the cell surface proteome generated by Bausch-Fluck *et al.*^27^

The E14TG2a data set we present acts both as a resource for interrogating the subcellular location of proteins of interest to researchers, and also acts as a scaffold onto which other high content data sets may be mapped to assist in their interpretation. HyperLOPIT is a powerful tool for gaining insights into fundamental post-translational processes governing stem cell behaviour.

## Methods

### Cell culture

Murine pluripotent ES cells (cell line E14TG2a) a kind gift from Professor Austin Smith, University of Cambridge, and available from American Type Culture Collection (CRL-1821), were maintained in culture on gelatinized flasks in a media containing fetal bovine serum supplemented with leukaemia inhibitory factor (LIF), as previously described^60^. Approximately 10^8^ cells were collected by trypsinization and washed several times with phosphate buffered saline. Cell suspension (10%) was aliquoted for chromatin extraction, while the remaining 90% was used for membrane fractionation.

### Immunocytochemistry

E14TG2A mES cells were plated and stained as described in ref. 42 using anti-GM130 (AbCam—EP829Y) and KDel (AbCam—ab50601), and imaged by confocal microscopy.

### Cell lysis and subcellular fractionation

For density-gradient ultracentrifugation, cell pellets were resuspended in 15 ml lysis buffer (0.25 M sucrose, 10 mM HEPES pH 7.4, 2 mM EDTA, 2 mM magnesium acetate) containing protease inhibitors (Roche), and lysed with a ball-bearing homogenizer (Isobiotec) on ice. Lysate viscosity was reduced by treatment with 25 U ml^−1^ benzonase endonuclease (Invitrogen) for 20 min at room temperature. Insoluble cellular debris was removed by centrifugation at 200*g*, 5 min at 4 °C. The supernatant was retained and the centrifugation step was repeated a further two times.

Optiprep density-gradient medium (60% w/v iodixanol, Sigma) was diluted to a working solution of 50% w/v iodixanol with 6 × lysis buffer (60 mM HEPES pH 7.4, 12 mM EDTA pH 8.0, 12 mM magnesium acetate), containing protease inhibitors. Solutions with varying concentrations of iodixanol were then made by mixing the iodixanol working solution and lysis buffer. The iodixanol concentration of each solution was verified by measuring refractive index using a handheld refractometer (Reichert Technologies).

The lysate was divided between several 5 ml polyallomer ultracentrifuge tubes (Beckman), and underlaid with 0.8 ml of 6% w/v iodixanol solution, and then with 0.8 ml of 25% w/v iodixanol solution. Samples were centrifuged in an Optima XL-80 ultracentrifuge (Beckman), SW55Ti rotor at 100,000*g*, 60 min at 4 °C. The resulting supernatant was retained as a ‘soluble fraction' (enriched in cytosolic proteins), and crude membranes were collected from the interface of the two iodixanol layers. The crude membrane fraction was diluted with lysis buffer and pelleted by ultracentrifugation in the SW55Ti rotor at 200,000*g*, 40 min at 4 °C to remove any residual cytosol from the membranes. The washed membrane pellet was then resuspended in 25% w/v iodixanol solution, and underlaid beneath a pre-formed gradient composed of 8, 12, 16 and 20% iodixanol layers, which was left for 8 h at 4 °C to diffuse to linearity. The continuous density gradient was centrifuged at 100,000*g* for 8 h in a VTi65.1 rotor at 4 °C with slow braking to minimize gradient disruption. Following ultracentrifugation, 20 × 0.5 ml gradient fractions were collected using an Auto Densi-Flow peristaltic pump with meniscus tracking probe (Labconco). The refractive indices of all fractions were measured to determine the shape of the final gradient. Each fraction was then diluted with 0.8 ml lysis buffer, and centrifuged in a TLA-55 fixed angle rotor at 180,000*g* in an Optima MAX-XP benchtop ultracentrifuge (Beckman), 20 min at 4 °C. This centrifugation step was repeated for all fractions, the supernatant was discarded and the resulting membrane-enriched pellets were stored at −20 °C. Four volumes of chilled acetone were added to the cytosolic fraction and the chromatin-enriched fraction, and protein precipitation was carried out overnight at −20 °C. The acetone samples were centrifuged and air-dried before solubilization in 8 M urea, 0.1% SDS, 25 mM tetraethylammonium bromide (TEAB; pH 8.5). The density-gradient membrane-enriched pellets were also resuspended in 8 M urea buffer. Samples were briefly sonicated on ice to ensure re-solubilization.

The reproducibility of gradients from three independent biological replicates can be found in Supplementary Table 2.

### Chromatin extraction and enrichment

Chromatin extracts were prepared as previously described^61^. Briefly, cells were resuspended in chromatin buffer A (10 mM HEPES pH 7.9, 10 mM KCL, 1.5 mM MgCl_2_, 0.34 M sucrose, 10% glycerol, 1 mM dithiothreitol) with protease inhibitors. Triton X-100 was added to a concentration of 0.1% v/v and incubated on ice for 8 min to lyse the cells. Nuclei were pelleted by centrifugation at 1,300*g*, 5 min at 4 °C. The nuclear pellet was resuspended in chromatin buffer B (3 mM EDTA, 0.2 mM EGTA, 1 mM dithiothreitol) with protease inhibitors and incubated for 30 min on ice. Samples were then centrifuged at 1,700*g*, 5 min at 4 °C. The chromatin-enriched pellet was washed in chromatin buffer B, re-pelleted and stored at −20 °C.

### Protein digestion and TMT 10-plex labelling

Protein concentrations were determined by BCA assay (Thermo Fisher Scientific) as per the manufacturer's instructions. Optimal fractions were selected for TMT 10-plex labelling based on western blot evaluation of organelle marker proteins, protein concentration and refractive indices of membrane fractions. Protein (50 μg) from 10 differentially enriched subcellular fractions (8 membrane fractions from the density gradient, plus cytosol and chromatin-enriched fractions, Supplementary Table 3) was reduced, alkylated and digested with trypsin. Briefly, each sample was made up to a total volume of 50 μl with 25 mM TEAB and reduced and alkylated. Disulfide bonds were reduced with 5 μl of 200 mM tris(2-carboxyethyl)phosphine, 1 h at 37 °C, followed by alkylation of cysteine residues with 5 μl of 375 mM iodoacetamide, 30 min at room temperature. Samples were then diluted tenfold with 25 mM TEAB and digested with sequencing grade trypsin (Promega) for 1 h with a 1:40 enzyme:protein ratio, 37 °C. An additional aliquot of trypsin at 1:40 concentration was added and incubated overnight at 37 °C. Trypsin digests were centrifuged for 10 min at 13,000*g* to remove any insoluble matter, then reduced to dryness by vacuum centrifugation.

While the TMT tags were equilibrating to room temperature, peptide samples were resuspended in 30 μl 1 M TEAB and 70 μl isopropanol. The solubilized samples were transferred into the tag vials and placed on a shaker for 2 h at room temperature. The reaction was quenched by addition of 8 μl 5% hydroxylamine for 30 min. The labelled samples were then combined and reduced to dryness by vacuum centrifugation. C18 solid-phase extraction was performed using Sep-Pak cartridges (100 mg bed volume, Waters) and peptides were eluted in 70% acetonitrile+0.05% acetic acid. The eluate was again reduced to dryness by vacuum centrifugation, and resuspended in 20 mM ammonium formate (pH 10.0), for high pH reversed-phase liquid chromatography.

### Sample fractionation

Desalted peptides were resuspended in 0.1 ml 20 mM ammonium formate (pH 10.0)+4% (v/v) acetonitrile. Peptides were loaded onto an Acquity bridged ethyl hybrid C18 UPLC column (Waters; 2.1 mm inner diameter × 150 mm, 1.7 μm particle size), and profiled with a linear gradient of 5–60% acetonitrile+20 mM ammonium formate (pH 10.0) over 60 min, at a flow rate of 0.25 ml min^−1^. Chromatographic performance was monitored by sampling eluate with a diode array detector (Acquity UPLC, Waters) scanning between wavelengths of 200 and 400 nm. Fractions were collected at 1 min intervals. Twenty-four fractions representing peak peptide elution were selected for mass spectrometry analysis and resuspended in 0.05% trifluoroacetic acid. Approximately 1 μg peptides were loaded per liquid chromatography-mass spectrometry run.

### Mass spectrometry

All mass spectrometry experiments were performed on an Orbitrap Fusion coupled with a Proxeon EASY-nLC 1000 (Thermo Fisher Scientific). Peptides were separated on a Proxeon EASY-Spray column (Thermo Scientific; 50 cm × 75 μm inner diameter, 2 μm particle size and 100 Å pore size). Separation was achieved by applying a 5–25% gradient of acetonitrile+0.1% formic acid over 95 min at 300 nl min^−1^, followed by 25–40% acetonitrile+0.1% formic acid over 10 min. An electrospray voltage of 1.8 kV was applied to the eluent via the EASY-Spray column electrode.

The Orbitrap Fusion was operated in positive ion data-dependent mode for both MS^2^ and SPS-MS^3^ methods. For the MS^2^ method, the full scan was performed in the Orbitrap in the range of 300–1,600 m/z at nominal resolution of 1.2 × 10^5^, followed by selection of the most intense ions above an intensity threshold of 2 × 10^4^ for high-energy collisional dissociation (HCD)-MS^2^ fragmentation. Ion filtering for MS^2^ events was performed by the quadrupole with a transmission window of 1.5 m/z. HCD fragmentation was performed with 40% normalized collision energy, followed by analysis of fragment ions in the Orbitrap with nominal resolution of 6 × 10^4^. The number of HCD-MS^2^ events between full scans was determined on-the-fly so that the duty cycle was fixed at 3 s.

The automatic gain control (AGC) settings were 4 × 10^5^ ions and 1 × 10^5^ ions, and maximum ion accumulation times to 50 and 120 ms, for full and MS^2^ scans, respectively. Ions with 1+ or undetermined charge state were excluded from MS^2^ selection. Ions within a ±10 p.p.m. m/z window around ions selected for MS^2^ were excluded from further selection for fragmentation for 35 s.

For the SPS-MS^3^ method, the full scan parameters were identical to those for the MS^2^ method. The most intense ions above a threshold of 2 × 10^4^ were selected for collision induced dissociation (CID)-MS^2^ fragmentation, with an AGC target and maximum accumulation time of 1 × 10^4^ and 70 ms. Mass filtering was performed by the quadrupole with 1.5 m/z transmission window, followed by CID fragmentation in the linear ion trap with 35% normalized collision energy. SPS was applied to co-select 10 fragment ions for HCD-MS^3^ analysis. SPS ions were all selected within the 400–1,000 m/z range, and were set to preclude selection of the precursor ion and TMTC ion series^62^. AGC targets and maximum accumulation times were set to 1 × 10^5^ and 120 ms. Co-selected precursors for SPS-MS^3^ underwent HCD fragmentation with 55% normalized collision energy, and were analysed in the Orbitrap with nominal resolution of 6 × 10^4^. The number of SPS-MS^3^ spectra acquired between full scans was restricted to a duty cycle of 3 s.

To assess the effect of using different numbers of precursors for SPS, ions were selected from full scans as described above. For each selected peptide ion, a sequence of six spectra was generated (conventional MS^2^, SPS with 15, 10, 5 and 2 precursors, and conventional MS^3^). The precursor ion for conventional MS^3^ was selected as the most intense ion within the 400–950 m/z range, excluding the unfragmented peptide and TMTC ion series, and isolated with a 2 m/z selection window. Conventional MS^2^ and SPS-MS^3^ were performed with the parameters described previously. The duty cycle for the sequence of scans was fixed at 6 s, with each sequence of six scans taking ∼1.5–2.5 s.

### Data processing

Raw data files were processed using Proteome Discoverer (v1.4, Thermo Fisher Scientific), interfaced with Mascot server (v2.3.02, Matrix Science). Mascot searches were performed against SwissProt mouse database (March 2013, 24,481 sequences), with carbamidomethylation of cysteine, and TMT 10-plex modification of lysine and peptide N termini set as modifications. For the MS^2^ method, in which identification was performed at high resolution in the Orbitrap, precursor and fragment ion tolerances of ±20 p.p.m. and ±0.2 Da were applied. For the SPS-MS^3^ method, in which identification was performed at lower resolution in the linear ion trap, tolerances of ±20 p.p.m. and ±0.5 Da were applied. Up to two missed tryptic cleavages were permitted. Searches were also performed against a sequence scrambled ‘decoy' database. The PSMs for the ‘forward' and ‘decoy' searches by Mascot were re-scored using the Percolator algorithm to yield a more robust false discovery rate^63^.

TMT 10-plex quantification was also performed by Proteome Discoverer by calculating the sum of centroided ions within ±2 mmu window around the expected m/z for each of the 10 TMT reporter ions. For SPS-MS^3^ methods, quantification was performed at the MS^3^ level. Spectra with more than four missing reporter ion values were excluded from quantification, and remaining missing values were set as zero for downstream analysis. For protein-level reporting, protein grouping was enabled, and values were calculated from the median of all quantifiable PSMs for each group. TMT values were then reported as a ratio to the sum of reporters in each spectrum (that is, the sum of the 10 values for each spectrum was equal to 1).

To evaluate the effect of the number of SPS notches on quantitative performance, data were first processed as described above. SPS data were then extracted from scan headers in the raw data files by a VB.NET script using MSFileReader libraries (Thermo Fisher Scientific). SPS data and reporter ion quantification were then paired with the peptide identification information reported by Proteome Discoverer.

### Machine learning and multivariate data analysis

The Bioconductor^64^ packages MSnbase^65^ and pRoloc^25^ for the R statistical programming language^66^ were used for handling of the quantitative proteomics data and the protein-localization prediction. We employed the use of the full pRoloc pipeline in which proteins are assigned to a subcellular localization using a multi-step analysis framework.

Using the pRoloc software^25^, an initial application of novelty detection^26^ was conducted to identify and confirm the presence of organelle clusters in an unbiased data-specific manner. The novelty detection analysis was run as described in Breckels *et al.*^26^ using the classic Gaussian ellipsoidal mixture models for multivariate data, a stringent 200 iteration run (*N*=200), and outlier detection testing at the 5% level (*P*=0.05). The minimum number of proteins per new phenotype cluster was set to 20 proteins (GS=20), to allow detection of small organelles and complexes. A set of well-known residents from three distinct organelle structure; the mitochondria, plasma membrane and ER, and from three well-known and abundant protein complexes; the proteasome and two ribosomal subunits, 40S and 60S, were used as initial input markers for the discovery analysis (Supplementary Table 4). These initial markers were manually curated using information from the UniProt database^67^, the Gene Ontology^68^ and the literature. From the nature of the experimental design it was known that nuclear structures existed within the data, however, nuclear markers were left unlabelled in the discovery analysis to allow an unbiased detection of any sub-nuclear clusters. Also, markers that cover the lysosome, peroxisome and endosome were also left unlabelled to obtain an unbiased data-specific confirmation of their presence. Supplementary Fig. 15 shows the results of the discovery analysis and Supplementary Tables 5 and 6 show the number of clusters identified and the final marker set to be used in protein classification. This final set contained 13 different subcellular structures; the mitochondria, ER, plasma membrane, lysosome, peroxisome, endosome, actin cytoskeleton, extracellular matrix, nucleus (non-chromatin), chromatin, proteasome, 40S and 60S ribosomal subunits and the cytosol which were defined through a careful manual search of the literature and though validation of clusters from phenotype discovery analysis. These curated marker lists are available in the pRoloc software.

A SVM classifier, with a radial basis function kernel, using class specific weights was used for classification of unassigned proteins to one of the 14 known classes. The weights used in classification were set to be inversely proportional to the subcellular class frequencies to account for class imbalance. Algorithmic performance of the SVM on the data set was estimated using stratified fivefold cross-validation (creating five test/train partitions), which features an additional cross-validation on each training partition to optimize free parameters, sigma and cost, via a grid search (as described in ref. 23). This process was repeated 100 times and the best cost and sigma parameters were chosen based on the best F1 score; the harmonic mean of precision and recall. The best sigma was 0.01, which controls the bandwidth of the Gaussian, and the best cost was 16, which controls the balance between adherence to the training data and predictive performance on future unknown examples. As different organelles reflect different SVM score distributions (Supplementary Fig. 16), scoring thresholds were calculated per subcellular niche and were set based on concordance with gene ontology annotation to attain a 5% FDR. Unassigned proteins were then classified to 1 of the 13 compartments according to the SVM prediction if greater than the calculated class threshold. See also Supplementary Note 1.

### Visualization and annotation of spatial proteomics data

To enable straightforward access to the data, we have developed the pRolocGUI application (http://bioconductor.org/packages/devel/bioc/html/pRolocGUI.html), which provides an interactive visualization interface for spatial proteomics data. It employs modern JavaScript technology that directly interacts with the R data using the shiny Web application framework for R (http://shiny.rstudio.com/). The interface enables users to visualize annotated spatial proteomics data with PCA and protein profile plots, search for proteins of interest and overlay protein complexes and functional networks onto the subcellular map. The application can be used to specifically explore our mouse pluripotent stem cell data online (https://lgatto.shinyapps.io/christoforou2015/), or can be installed locally to visualize data from any spatial proteomics experimental designs with or without any analysis with the pRoloc pipeline. Documentation and a series of online tutorial videos for pRolocGUI can be found at http://ComputationalProteomicsUnit.github.io/pRolocGUI/.

## Additional information

**Accession codes:** The mass spectrometry data have been deposited to the ProteomeXchange Consortium (http://proteomecentral.proteomexchange.org)^69^ via the PRIDE partner repository with the data set identifier PXD001279.

**How to cite this article:** Christoforou, A. *et al.* A draft map of the mouse pluripotent stem cell spatial proteome. *Nat. Commun.* 7:8992 doi: 10.1038/ncomms9992 (2016).

## Supplementary Material

Supplementary Information Supplementary Figures 1-18, Supplementary Tables 1-6, Supplementary Note 1 and Supplementary References

Supplementary Data Set 1 Supplementary spread sheet containing quantitative proteomics data
