# Supplementary material for: A draft map of the mouse pluripotent stem cell spatial proteome
Source: Nat Commun. 2016 Jan 12;7:9992. doi: 10.1038/ncomms9992 (PMC4729960; doi:10.1038/ncomms9992)
Supplement: Supplementary Information — Supplementary Figures 1-18, Supplementary Tables 1-6, Supplementary Note 1 and Supplementary References [file ncomms9992-s1.pdf]

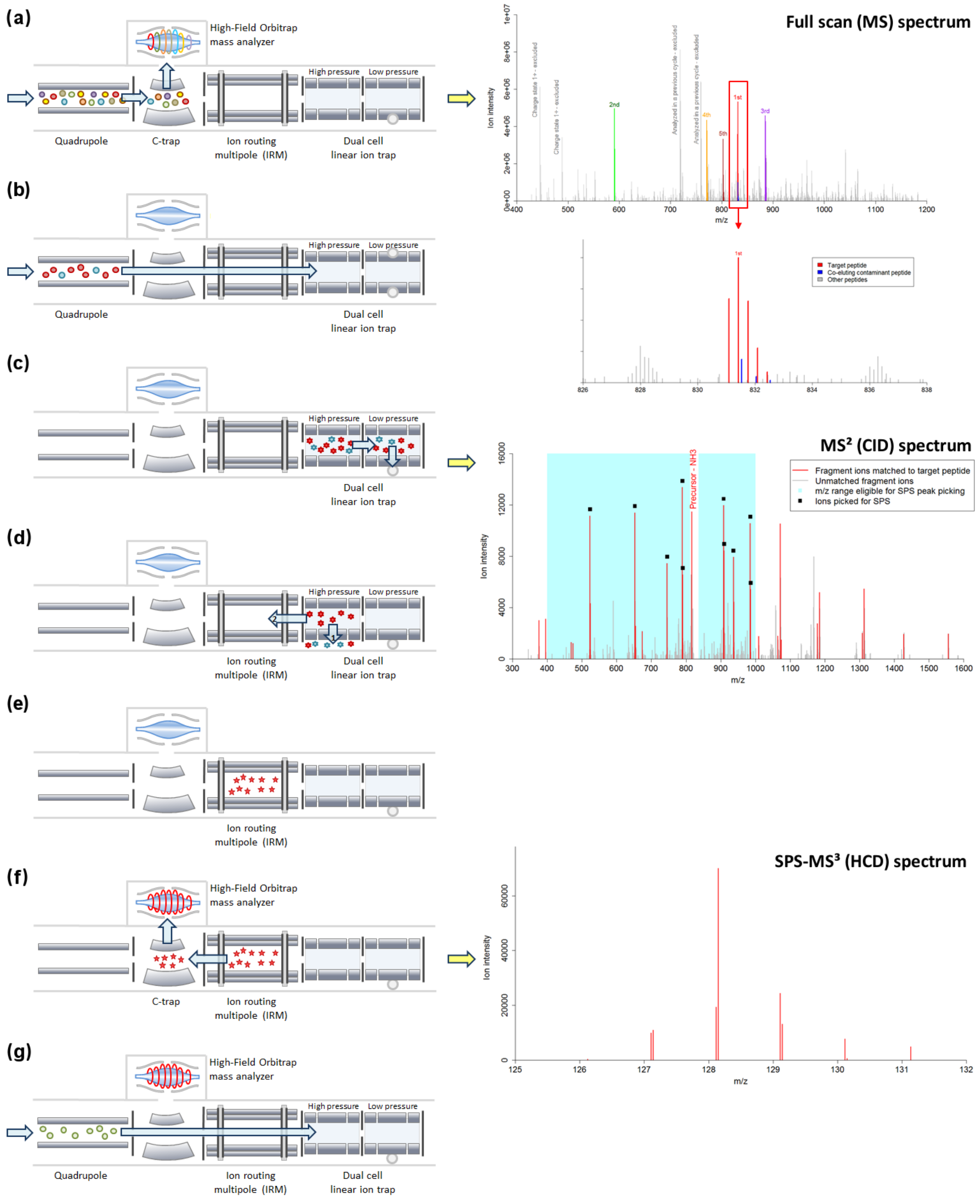
(a)
High-Field Orbitrap mass analyzer
High pressure
Low pressure
Quadrupole
C-trap
Ion routing multipole (IRM)
Dual cell linear ion trap
(b)
(c)
(d)
(e)
(f)
(g)
Full scan (MS) spectrum
Ion intensity
m/z
Charge state 1+ - excluded
Analyzed in a previous cycle - excluded
1st
2nd
3rd
4th
5th
Target peptide
Co-eluting contaminant peptide
Other peptides
MS² (CID) spectrum
Fragment ions matched to target peptide
Unmatched fragment ions
m/z range eligible for SPS peak picking
Ions picked for SPS
Precursor - NH3
SPS-MS³ (HCD) spectrum

Supplementary Figure 1 | Overview of steps performed in SPS-MS^3 acquisition. (a) Peptides eluting from the LC system undergo electrospray ionization. Cations are then transmitted through a quadrupole and accumulated in a C-trap, before being passed into the Orbitrap mass analyzer to generate a full scan (MS) spectrum. The most intense ions detected in the full scan (5 in this example) are selected for SPS-MS^3 analysis. (b) The first precursor selected in the full scan (red) is isolated and transmitted to the linear ion trap via the ion routing multipole (IRM). Due to the high sample complexity, other peptides with similar *m/z* and LC retention properties are co-selected (blue). (c) Peptides are fragmented by collision induced dissociation (CID) in the high pressure cell of the ion trap. Fragments are then transferred to the low pressure cell and scanned out onto two electron multipliers to generate an MS^2 CID spectrum. The most intense ions in this spectrum that meet specified criteria (as described in the Methods section), which are typically specific to the target peptide, are determined on-the-fly and picked for SPS. The MS^2 spectrum is also used post-acquisition for peptide identification. (d) Steps b and c are repeated for the same precursor. Rather than transferring peptide fragments to the low pressure cell after fragmentation, an SPS isolation waveform is applied to eject all ions from the linear ion trap except those chosen for SPS (1). The purified SPS ions are then transferred to the IRM (2). (e) The SPS ions undergo a second round of fragmentation by higher-energy collisional dissociation (HCD), liberating TMT reporter ions from the labeled peptide fragments. (f) These fragments are transferred via the C-trap into the Orbitrap to generate an SPS-MS^3 HCD spectrum, which is used for relative quantification of TMT 10-plex reporter ions in the *m/z* range of 126-131. (g) In order to utilize analysis time efficiently, operation of the Orbitrap and linear ion trap is parallelized. For example, while the Orbitrap is acquiring the SPS-MS^3 for the first precursor (red), the second precursor (green) is already being isolated by the quadrupole and fragmented in the linear ion trap to generate its MS^2 spectrum.

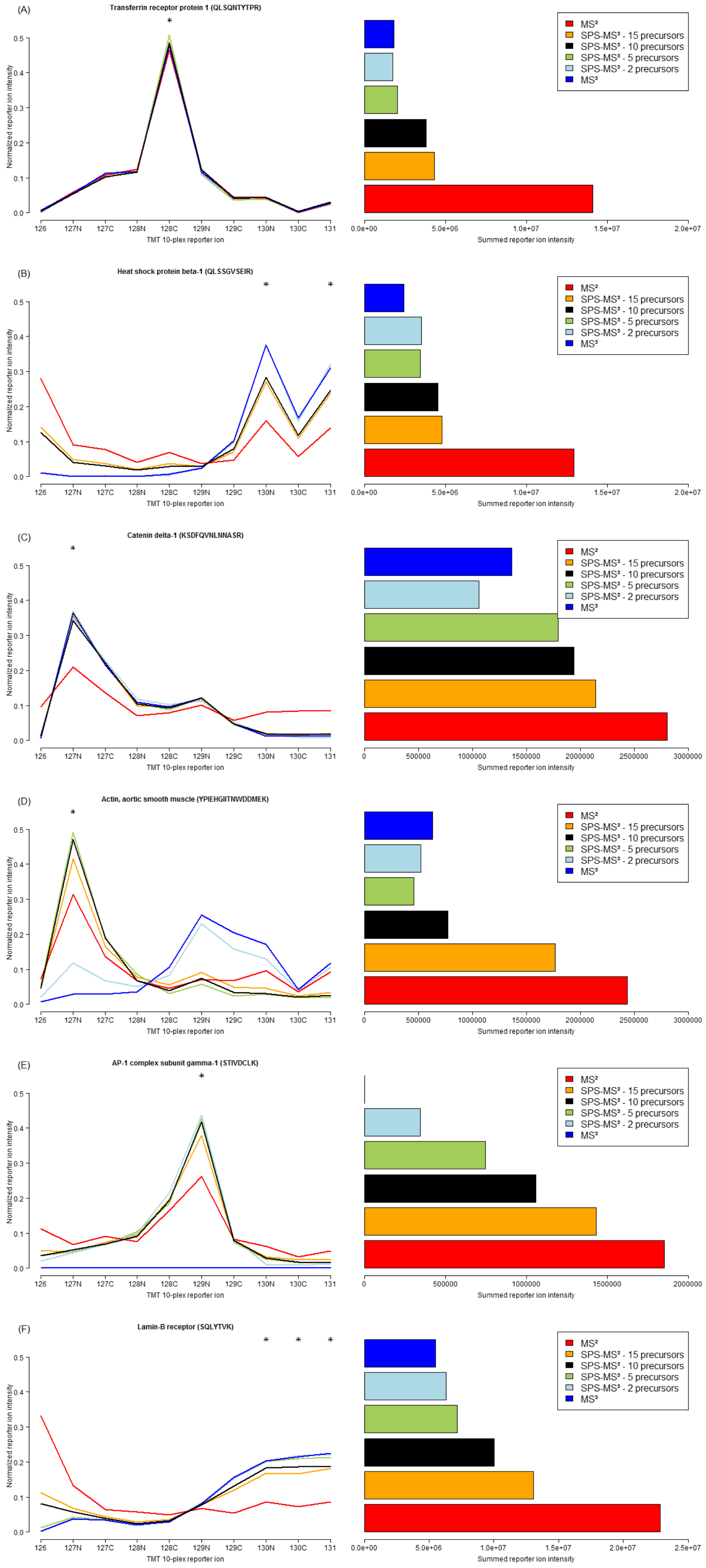

Supplementary Figure 2 | Examples of the differences observed with conventional MS^2, conventional MS^3, and SPS-MS^3 acquisition for Experiment 1. Plots on the left side demonstrate differences in quantitative accuracy, with the expected peak fraction(s) for a particular peptide represented by asterisks. Plots on the right show the effects of acquisition mode on summed reporter ion signal. (A) No change in quantitative performance observed. (B) SPS-MS^3 provides quantitative accuracy between that of MS^2 and MS^3, with fewer precursors producing more accurate data. (C) SPS-MS^3 and conventional MS^3 are both more accurate than MS^2, and SPS-MS^3 improves reporter ion signal relative to MS^3. (D) MS^3 and SPS-MS^3 with few precursors yield inaccurate quantification due to the contaminant fragment ion(s) being selected for re-fragmentation. SPS-MS^3 with more precursors compensates for this. (E) Conventional MS^3 is unquantifiable due to poor signal. SPS-MS^3 restores signal whilst preserving gains in quantitative performance over MS^2. (F) Distorted MS^2 reporter ion distribution results in misclassification of protein localization. The expected distribution is restored by SPS-MS^3.

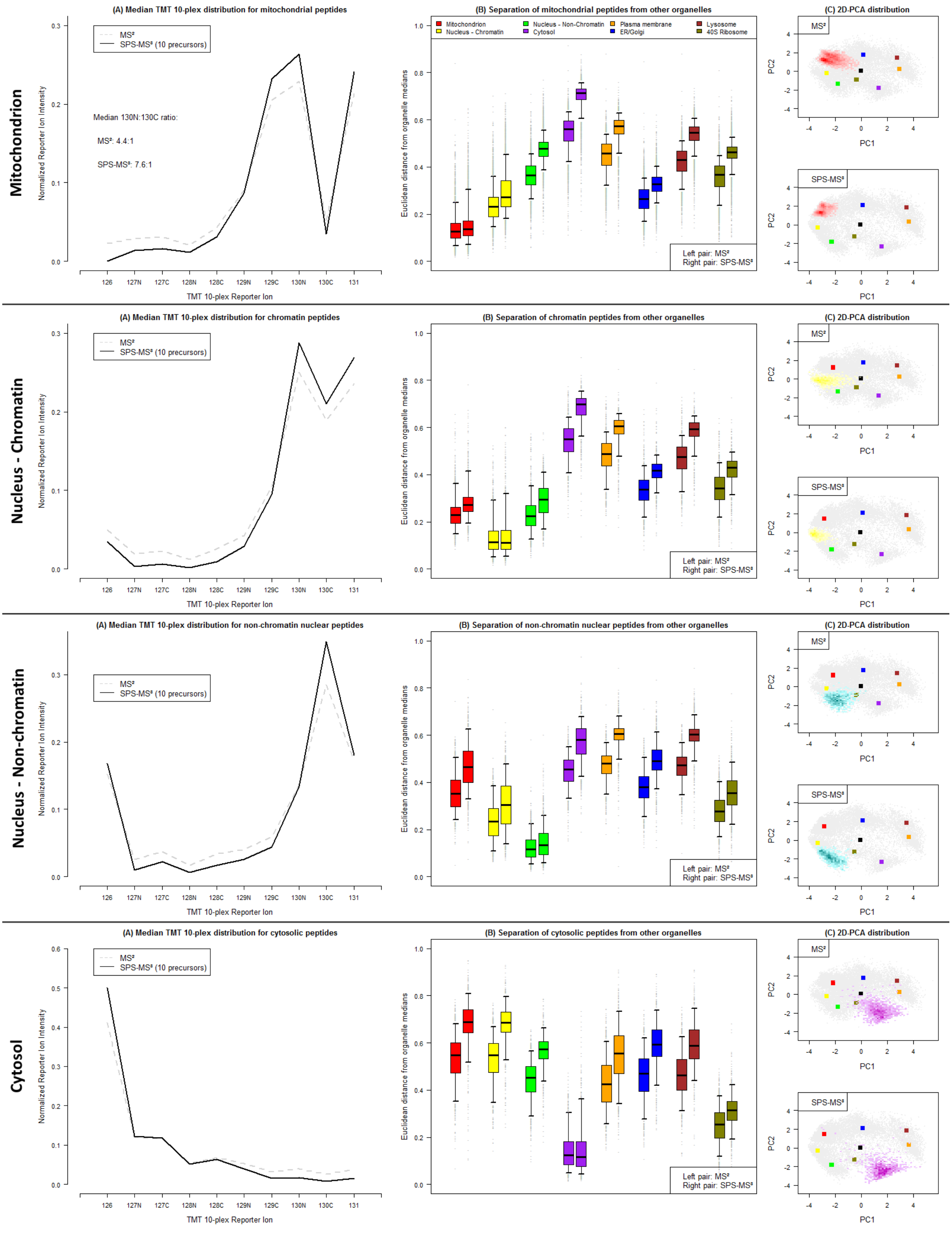

Supplementary Figure 3 | Enhanced organelle resolution with SPS-MS^3 relative to conventional MS^2 acquisition. Additional examples of the effect of SPS-MS^3 on organellar resolution to those presented in Figure 2.

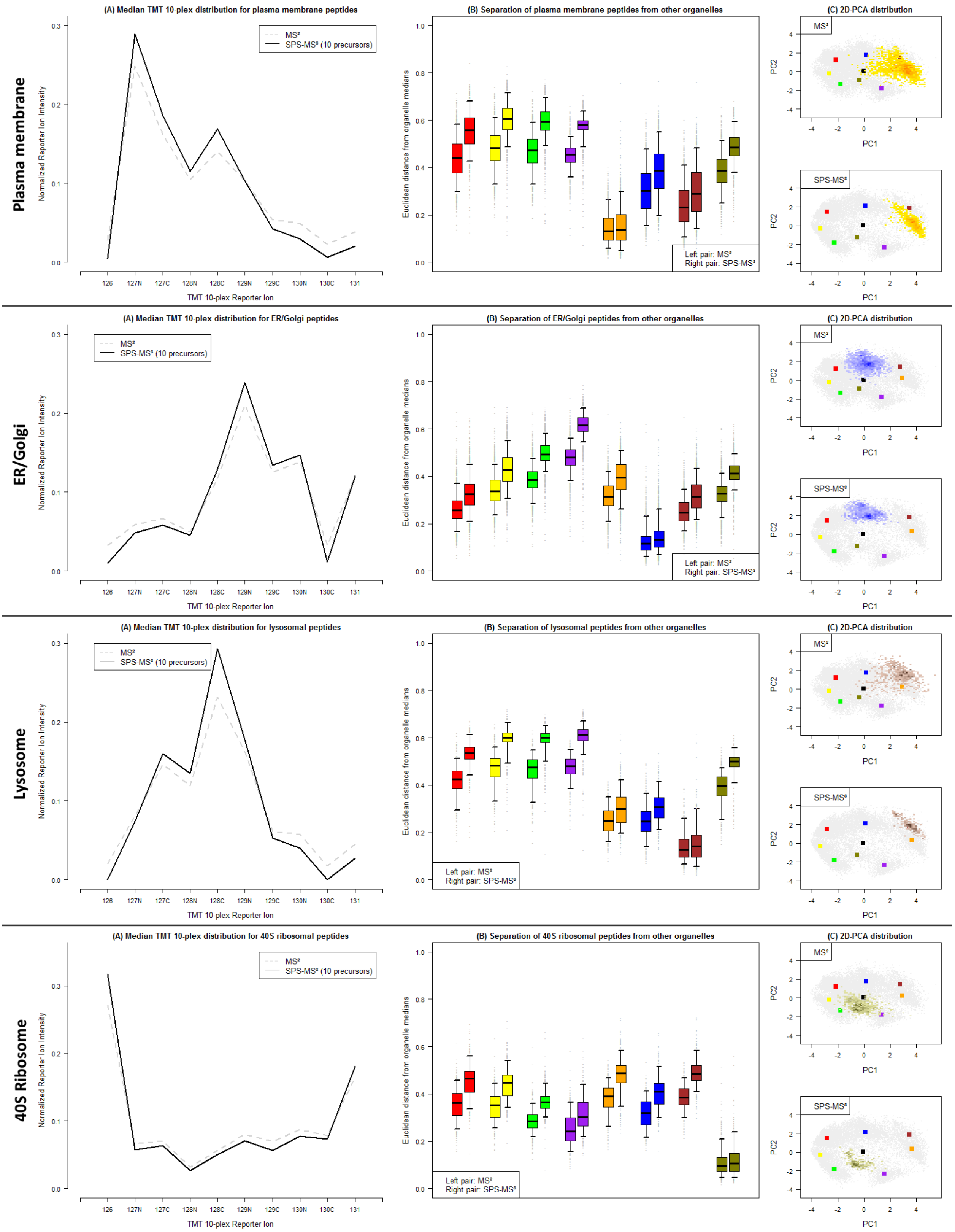

Supplementary Figure 3 (continued).

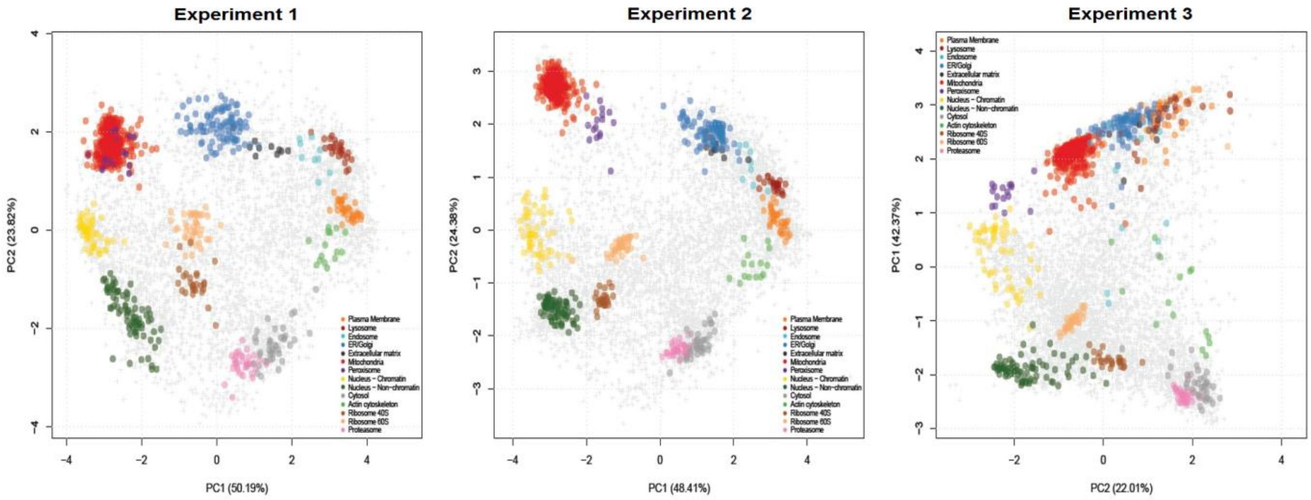

Supplementary Figure 4 | Principal component analysis plots for the three fractionation experiments. Organellar marker proteins are colored points, while other proteins appear as gray points.

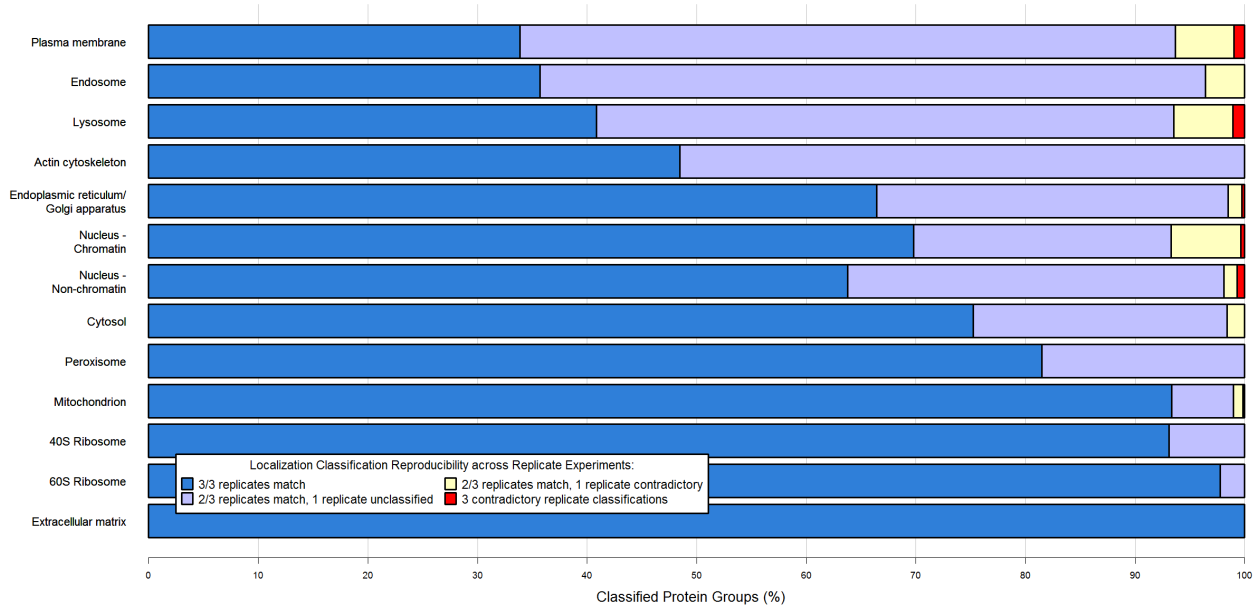

Supplementary Figure 5 | Concordance in protein localization assignments across three experiments. SVM classification was performed on each of the three experiments individually. 2,841 proteins were both identified in all three experiments, and assigned to an organelle class in at least 2 of 3 experiments. Over 90% of these proteins were either assigned to the same localization class in all 3 cases, or in 2 of 3 cases with the remaining experiment not classifying the protein to any of the 13 subcellular classes. Contradictory localization assignments occur at suitably low frequency for all organelle classes.

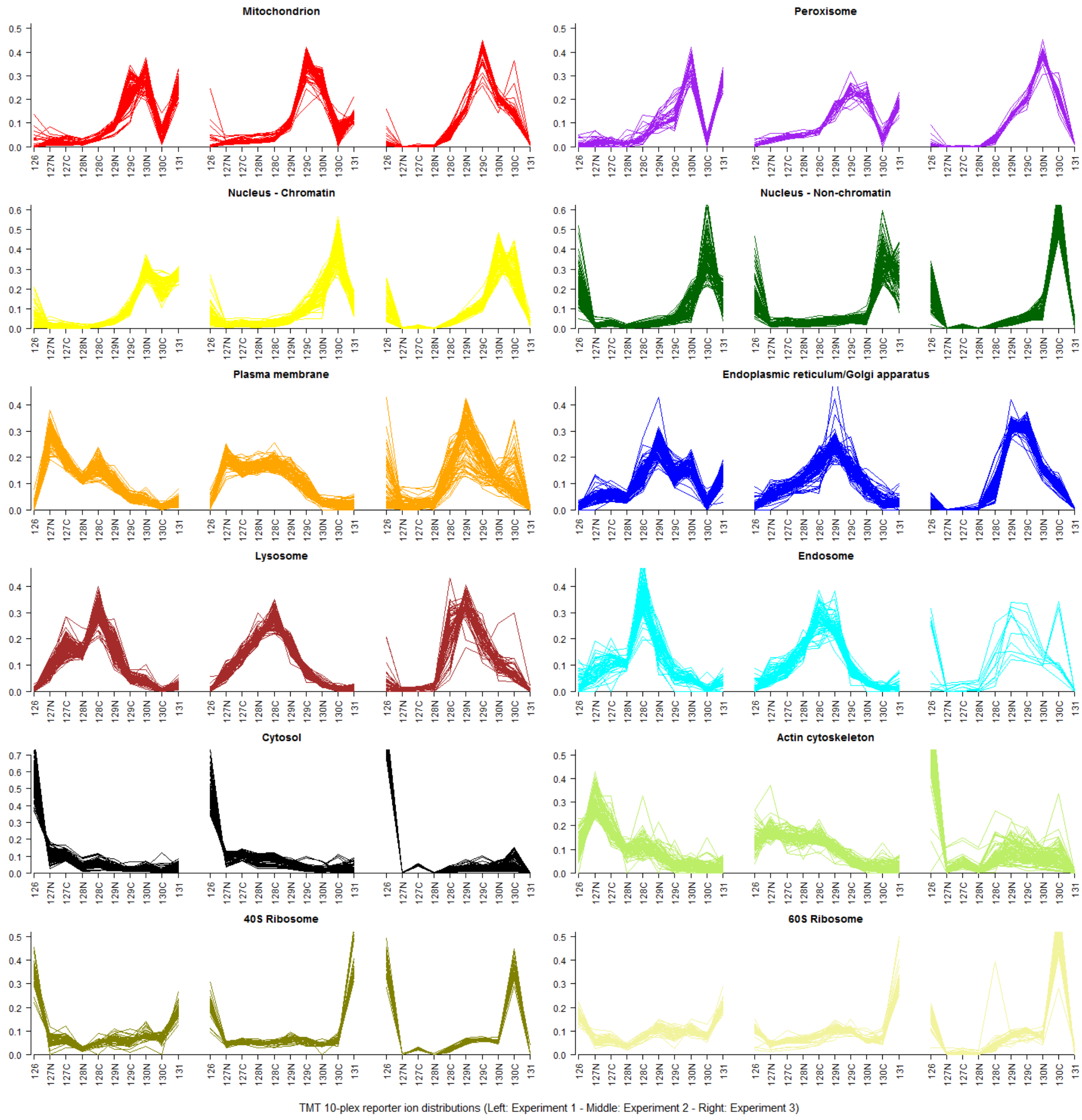

Supplementary Figure 6 | TMT 10-plex reporter ion distributions for two biological replicates of E14TG2a hyperLOPIT data. Proteins with the same steady-state subcellular localization co-distribute across the fractionation scheme and therefore show correlated and characteristic multivariate profiles.

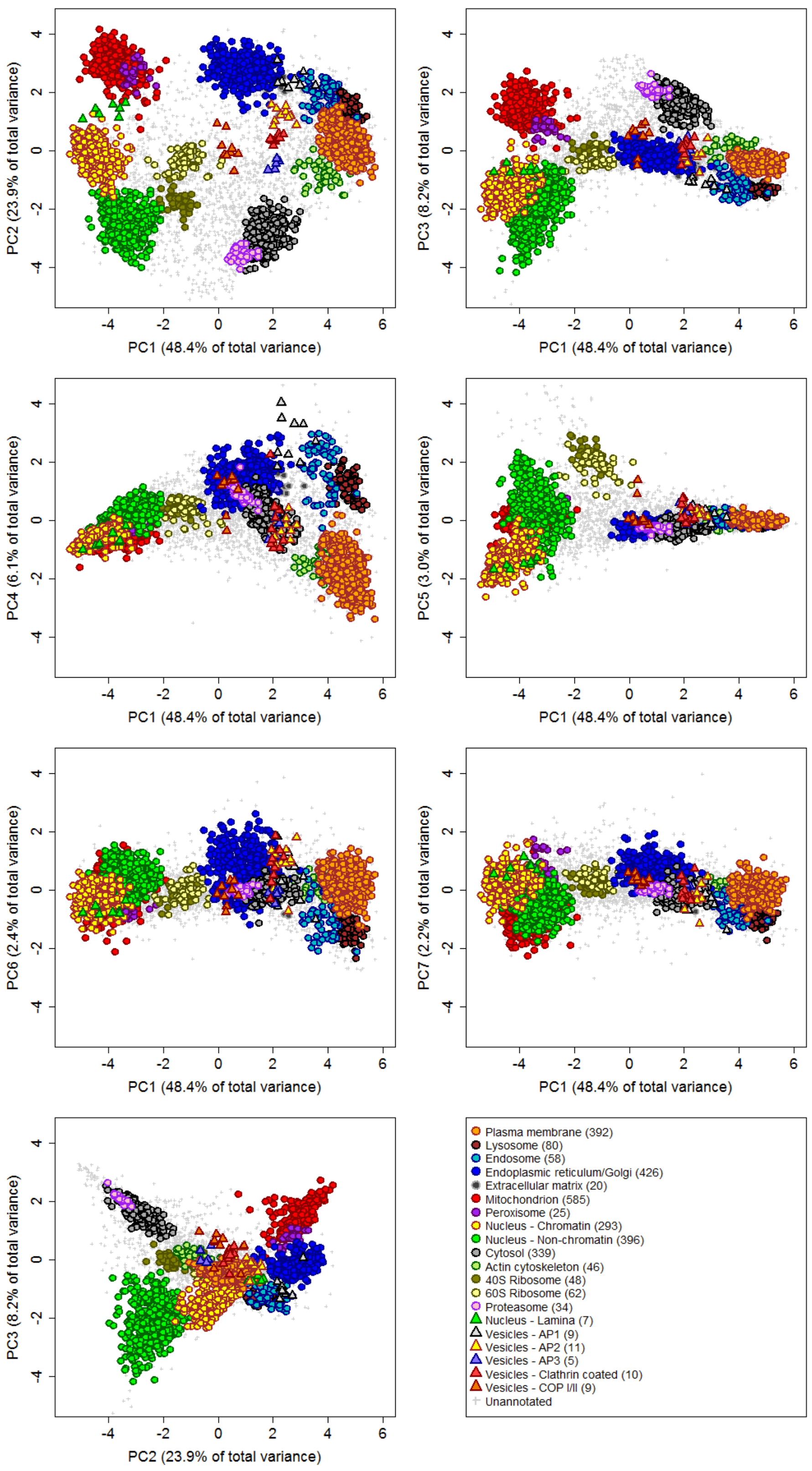

Supplementary Figure 7 | Lower principal components for hyperLOPIT data presented in Figure 3. Resolution of some compartments is more evident in the lower components, for example separation of plasma membrane and lysosome in PC3, and mitochondrion and peroxisome in PC7.

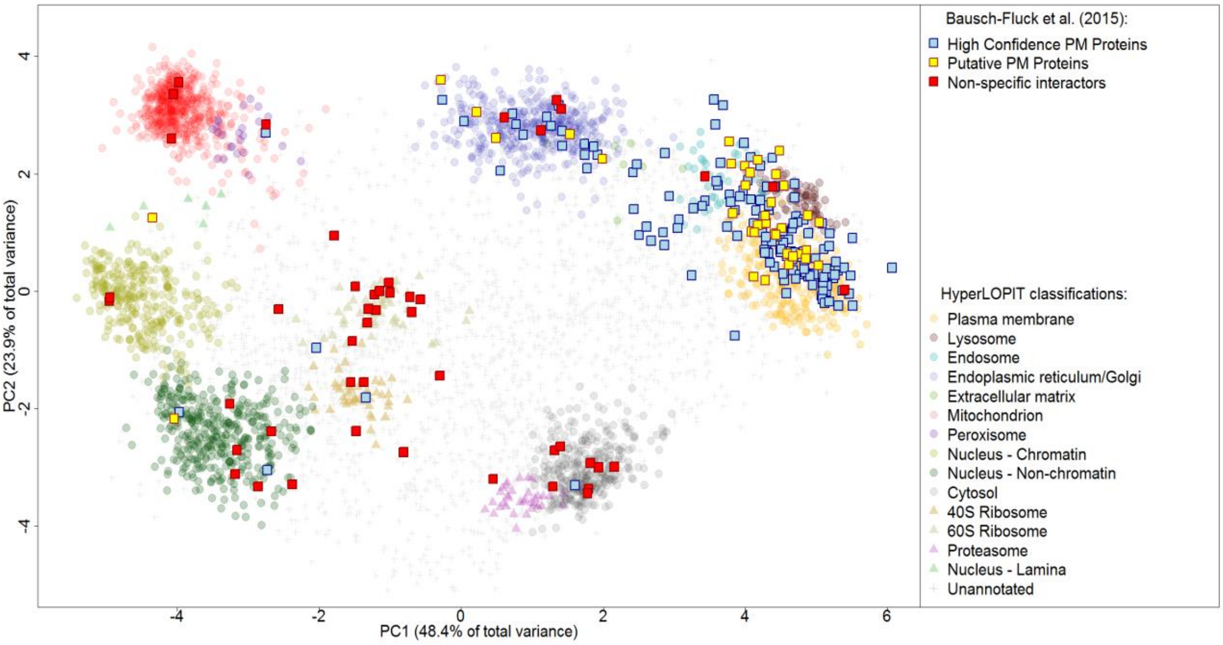

Supplementary Figure 8 | E14Tg2a cell surface capture data from Bausch-Fluck *et al.* [1] overlayed onto the hyperLOPIT dataset. HyperLOPIT verifies localization of high confidence cell surface proteins assigned by Bausch-Fluck and co-workers, and provides experimental evidence to support putative cell surface proteins. Most such proteins observed in the plasma membrane or trans-Golgi network in the hyperLOPIT data. Proteins described as non-specific interactors by Bausch-Fluck and co-workers display hyperLOPIT distributions that are inconsistent with cell surface proteins, such as mitochondrial, nuclear, ribosomal and cytosolic localization.

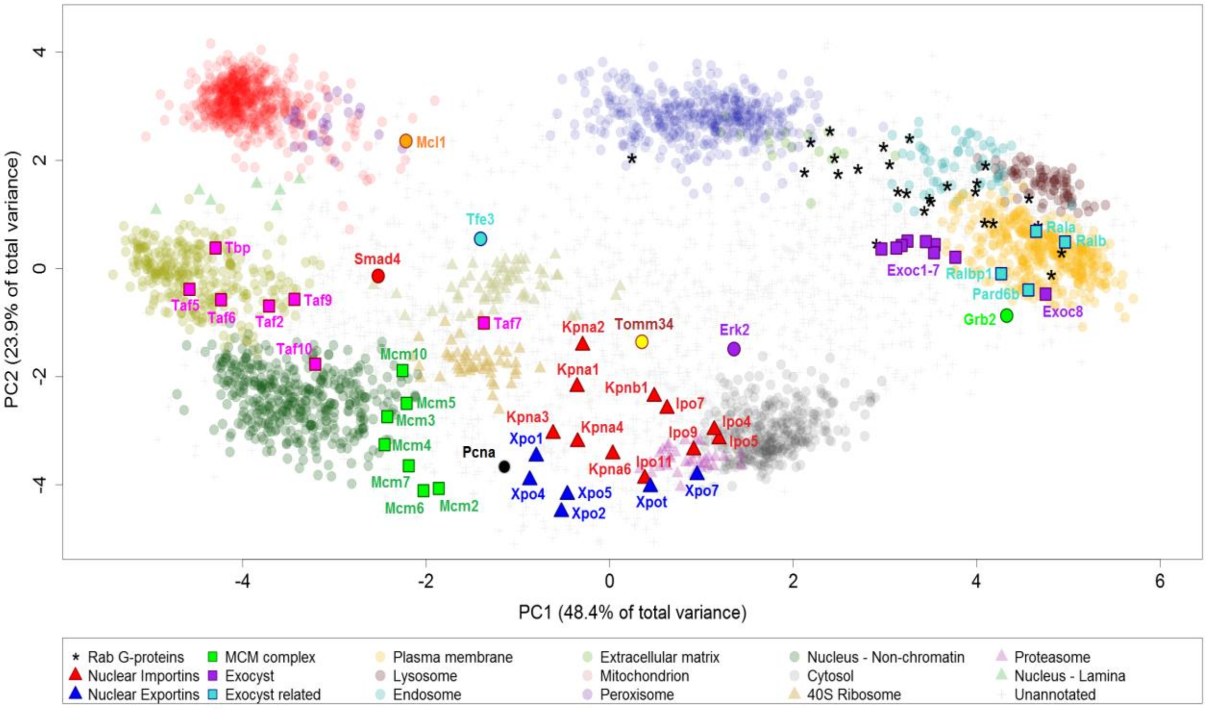

Supplementary Figure 9 | Examples of proteins displaying mixed localization. Proteins with mixed localization do not co-distribute with classifiable organelle phenotypes (muted colors), and therefore display characteristic distribution patterns. The nuclear import/export machinery demonstrates an intermediate position between the cytosol and the nucleus, while the Rab G-proteins are distributed throughout the secretory pathway. Similarly, the MCM (minichromosome maintenance) complex has a distinct location between the nucleus and cytoplasm, in accordance with its role in DNA replication initiation [2]. Tfe3 is a transcription factor whose nuclear/cytoplasmic ratio is indicative of differentiation status [3], while nucleocytoplasmic re-localization of Pcna has previously been demonstrated to modulate differentiation of neutrophils [4]. The Bcl-2 family member Mcl-1 displays an intermediate position between the mitochondria, endoplasmic reticulum and the nucleus [5]. Tom34 is a cytosolic co-chaperone involved in mitochondrial protein import [6]. Also shown are two examples of complexes where a single member of the complex has a distinct localization from the core group (TFIID complex and the exocyst complex). Taf7 is thought to dissociate from the TFIID complex following initiation of transcription, and also has a role in the assembly of several other transcription pre-initiation complexes, which may explain its separate steady-state location [7]. Exoc8 is localized away from the core exocyst complex, and co-distributes with its known binding partners Par6 and RalA [8].

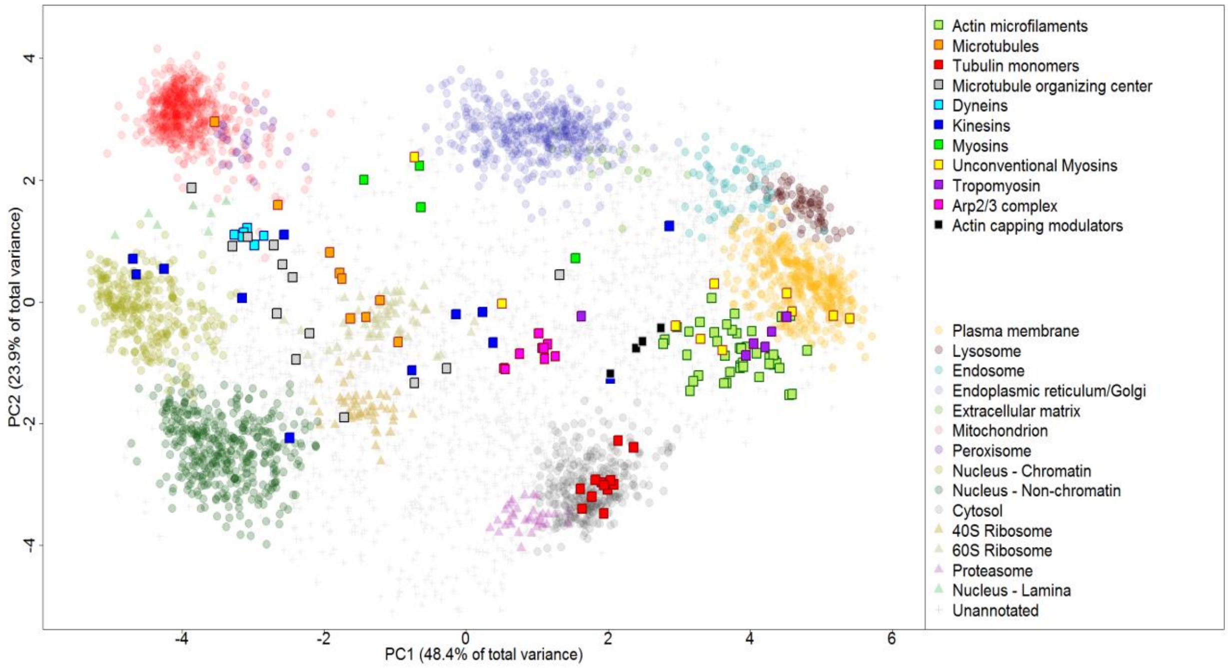

Supplementary Figure 10 | Fractionation of the cytoskeleton by hyperLOPIT. In addition to separating organelles and soluble proteins, cytoskeletal components also fractionate with characteristic distribution patterns.

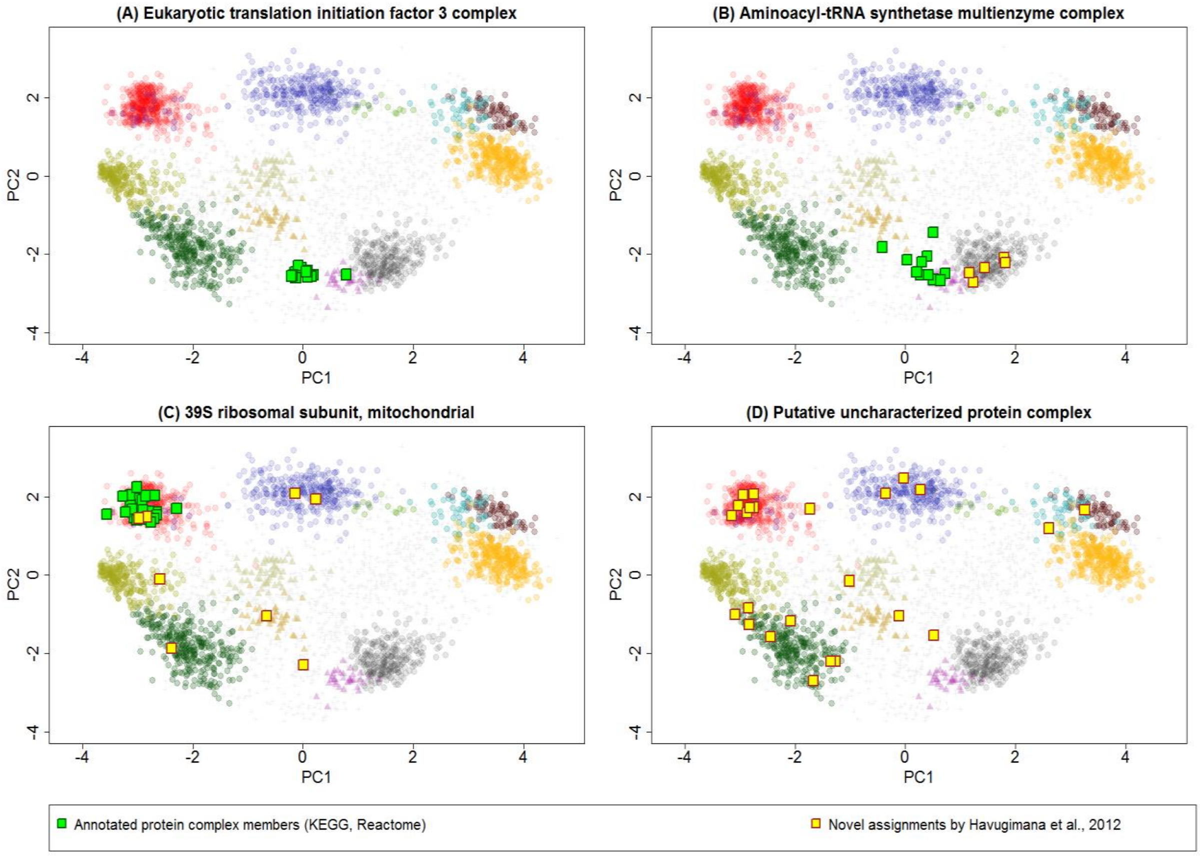

Supplementary Figure 11 | Mouse orthologues of protein complexes identified in a census of the human proteome. [9] (A) Components of highly curated and characterized complexes such as the eIF3 complex display closely correlated subcellular distribution profiles. (B) HyperLOPIT adds a spatial context to interactomics studies. The aminoacyl-tRNA synthetase complex is distributed between the cytosol and ribosome, consistent with its expected function. Additional assignments to the human orthologue of this complex by Havugimana and co-workers were localized to the cytosol, suggesting that their interaction with the complex is transient or unstable relative to the 'core' curated complex. (C) The spatial context can also be used to add additional confidence to novel assignments. Two of eight proteins novel assignments to the mitochondrial ribosome were found to localize to the mitochondrion and were therefore plausible interactors. The remaining six novel interactors were distributed across a range of other subcellular compartments, suggesting that these interactions are improbable. (D) Putative protein complexes can also be evaluated with this approach. Components of the putative complex shown here are distributed in many different subcellular compartments, suggesting that the probability of all components co-localizing to form a single complex is low. The putative complex is therefore likely a false positive in this case.

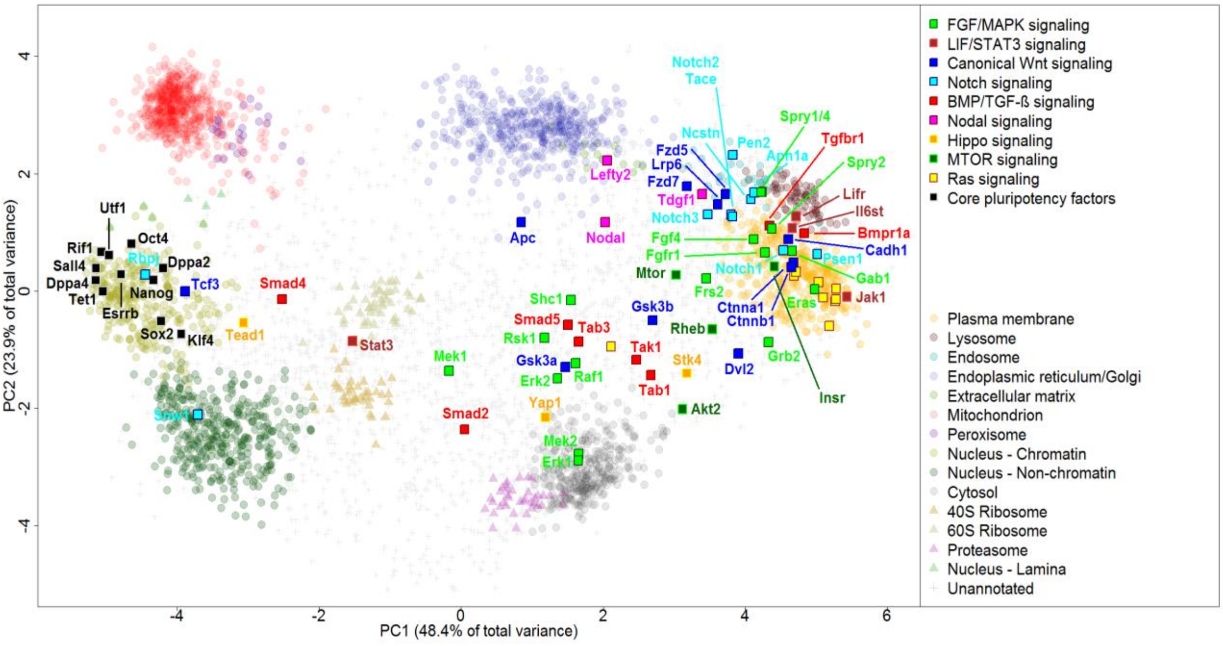

Supplementary Figure 12 | Subcellular localization of signaling cascade components. Pluripotency is maintained by a network of transcription factors which are influenced by several well-defined signaling pathways in combination with intrinsic and extrinsic factors. Six key signaling cascades involved in cell fate determination are highlighted, including cell surface receptors and ligands, intracellular adapters and transducers, and nuclear effectors.

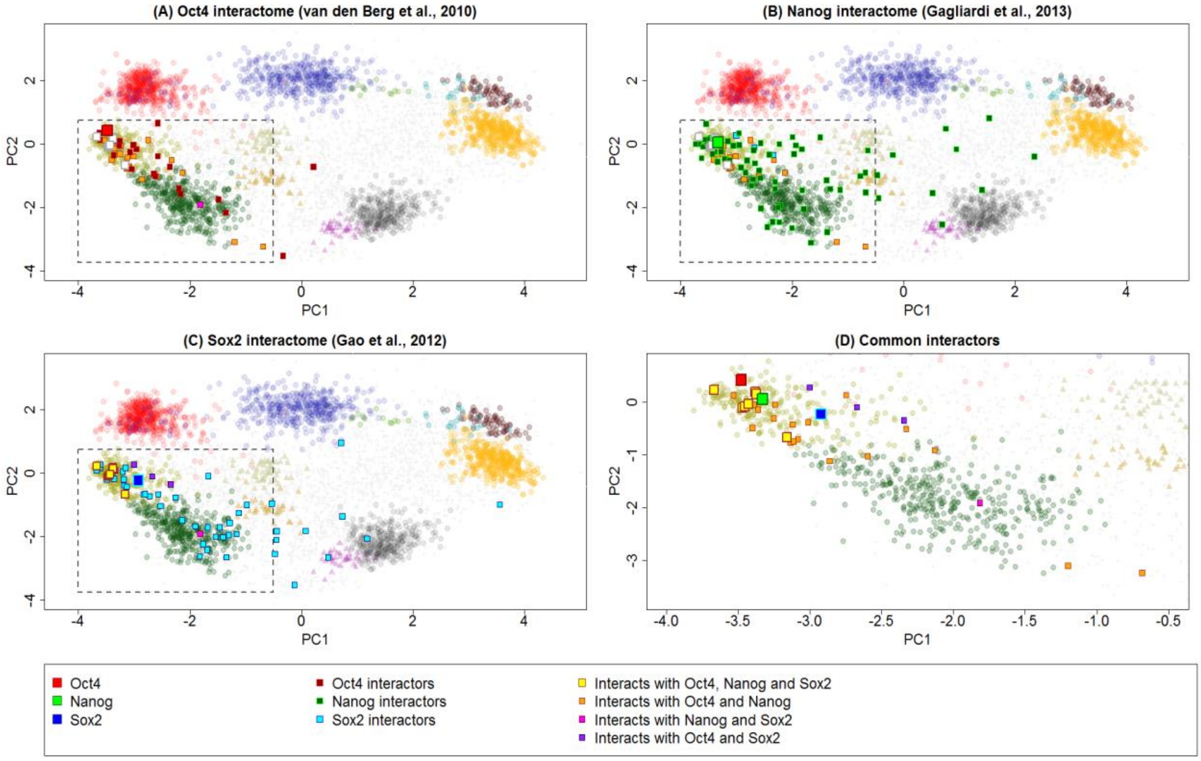

Supplementary Figure 13 | Spatial overview of the interaction partners of pluripotency triad Oct4, Nanog and Sox2. (A-C) HyperLOPIT reveals that while a majority of identified interaction partners of these three transcription factors are nuclear, some interaction partners were found with a variety of extranuclear distributions. (D) The shared interactors of the three transcription factors are predominantly localized to nuclear chromatin.[10] [11] [12]

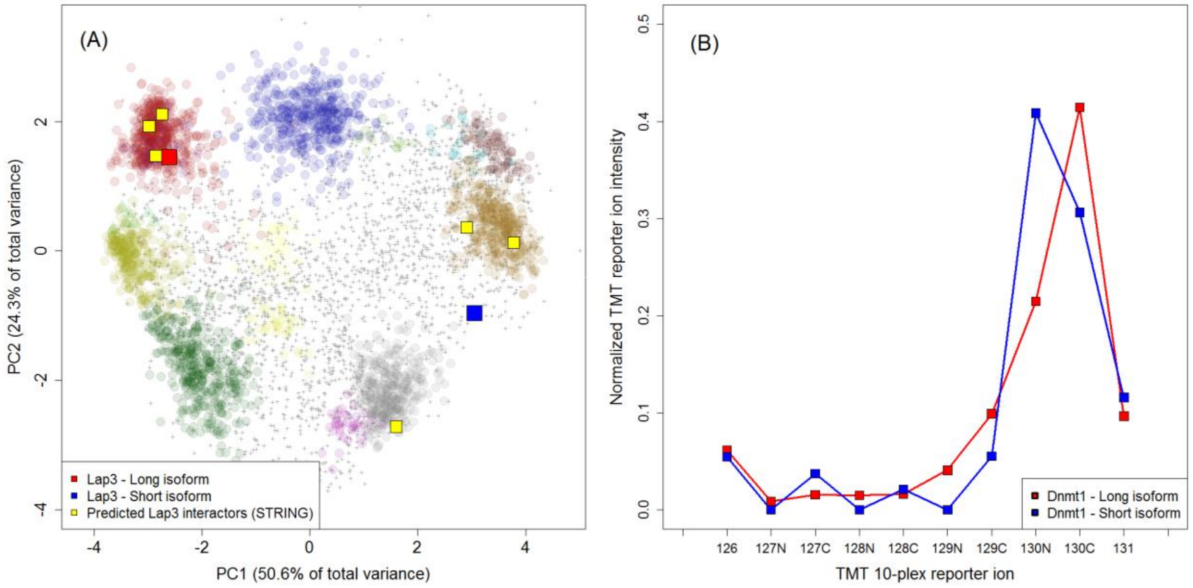

Supplementary Figure 14 | Examples of protein isoforms with differential subcellular localization in ES cells. (A) The long isoform of leucine aminopeptidase 3 (Lap3) was identified with mitochondrial localization, whereas the short isoform, which lacks the N-terminal import sequence, is localized between the cytosol and plasma membrane. Predicted interaction partners of Lap3 are found to localize across these three distributions, suggesting that the interactions are isoform-specific due to the differential compartmentalization of Lap3. (B) TMT 10-plex reporter ion profiles for the two isoforms of chromatin modifier Dnmt1 display differential localization. The long isoform enriches in the TMT 130C channel, consistent with chromatin localization, whereas the short isoform is most enriched in the TMT 129C channel, suggesting non-chromatin nuclear localization.

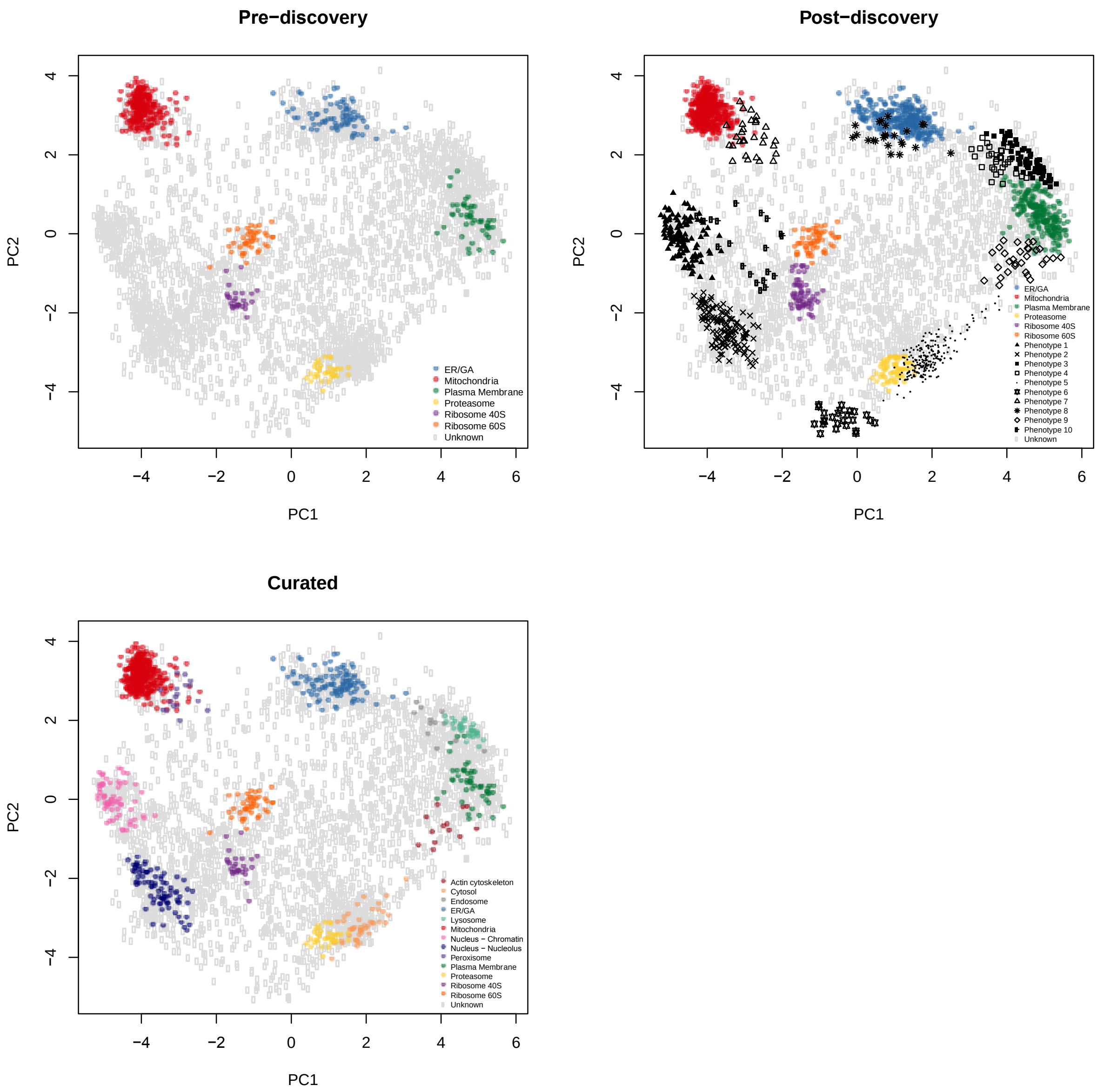

Supplementary Figure 15 | Application of novelty detection algorithm: initial marker proteins (top left), newly identified phenotypes (top right) and organelle markers after phenotype curation (bottom left).

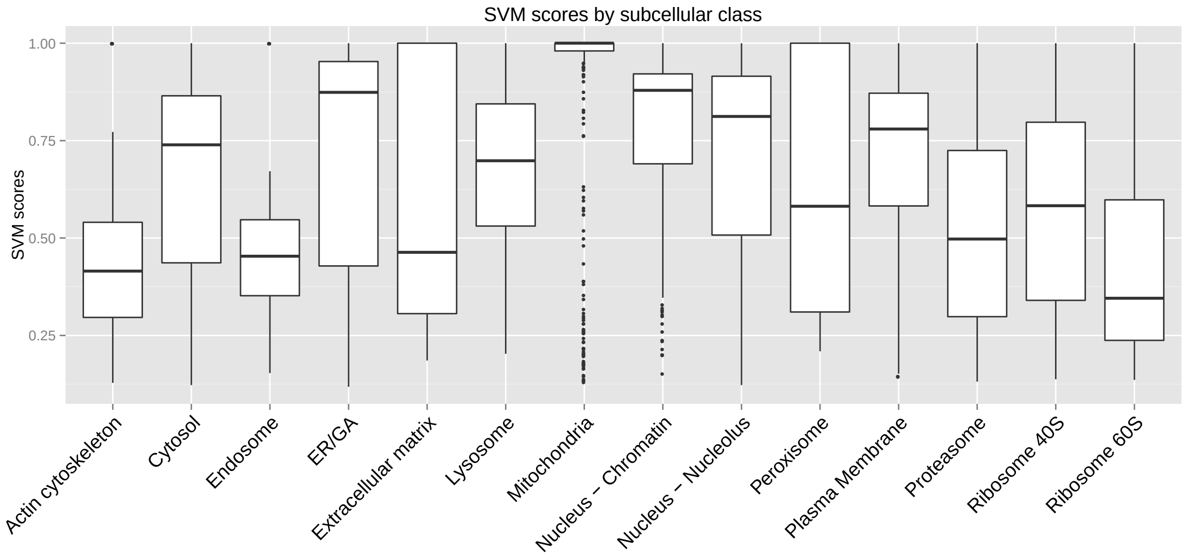

Supplementary Figure 16 | Organelle specific SVM classification score distributions. Choosing a global single threshold is not satisfactory as different subcellular niches exhibit different score distributions reflective of their resolution.

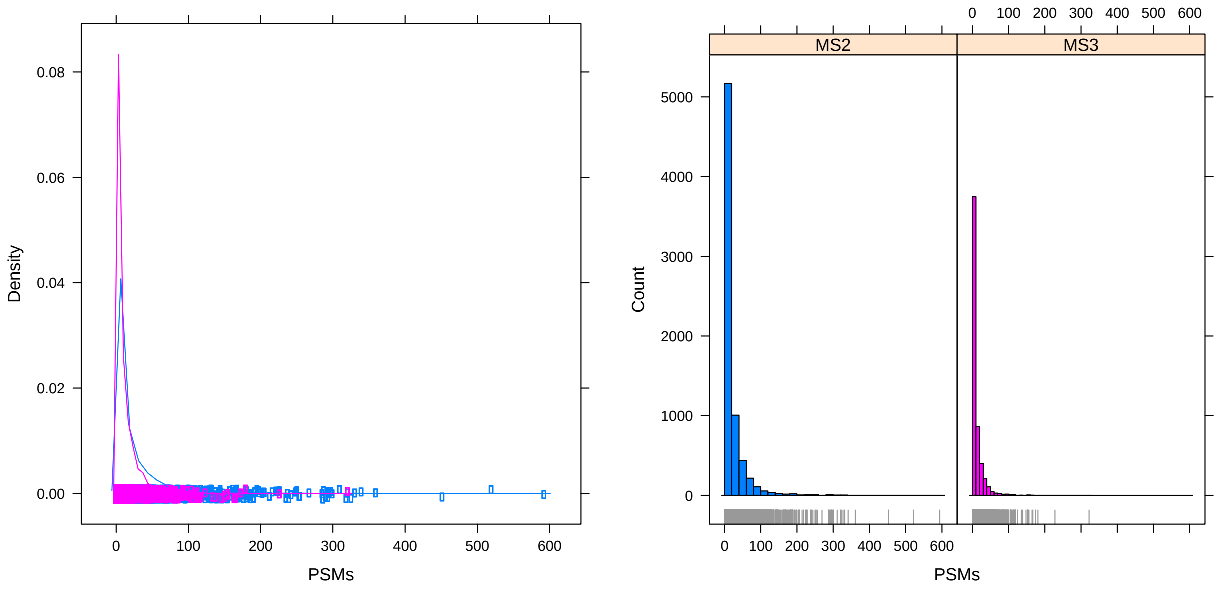

Supplementary Figure 17 | Histograms (density, left and absolute counts, right) for the number of PSMs per protein for the MS^2 (blue) and SPS-MS^3 (magenta) data illustrating the higher number of proteins and the higher number of PSMs per proteins in MS^2.

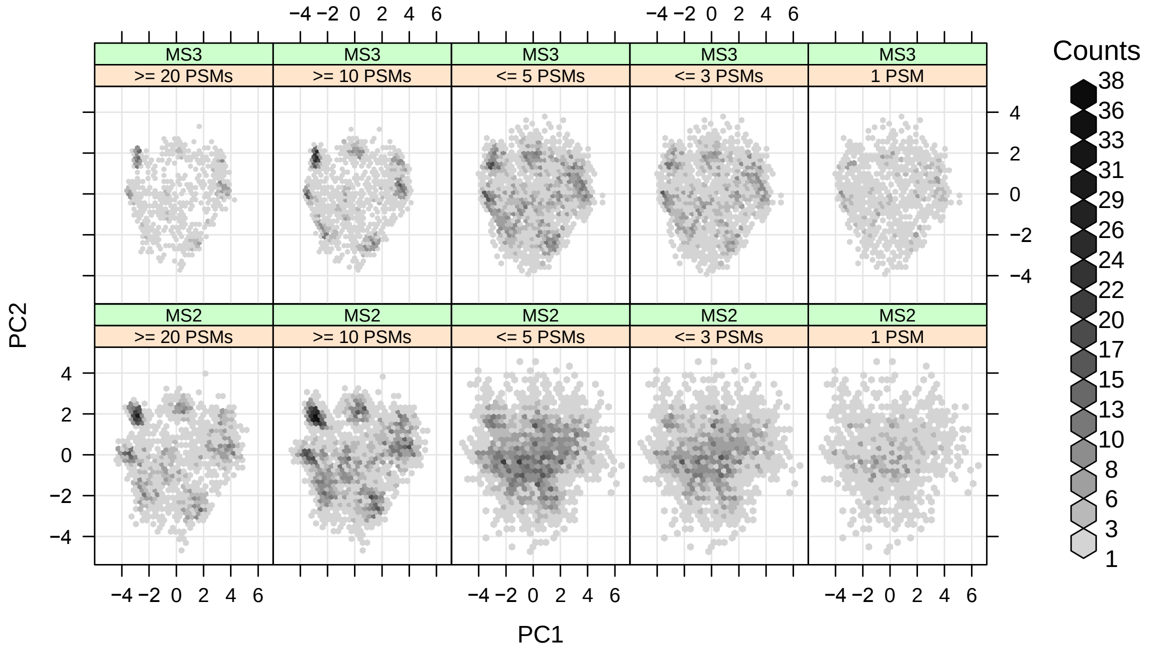

Supplementary Figure 18 | PCA plot densities for MS^2 and SPS-MS^3.

MS Data Acquisition Mode	SPS precursors	Peptide-spectrum matches (PSMs)	PSMs with summed reporter ion intensity > 1 x 10^5	PSMs with summed reporter ion intensity > 1 x 10^6	Median Summed TMT 10-plex Reporter Intensity	Mean Missing TMT 10-plex Values
**MS2**	-	4197	97.50%	79.50%	3.3 x 10^6	0.2
**MS3**	-	4161	74.90%	43.90%	6.8 x 10^5	2.1
**SPS-MS3**	2	4126	81.90%	49.80%	9.8 x 10^5	1.5
	5	4173	88.20%	57.40%	1.5 x 10^6	1.0
	10	4192	92.80%	65.80%	2.1 x 10^6	0.6
	15	4195	94.50%	70.50%	2.5 x 10^6	0.5

Supplementary Table 1 | Comparison of ion signal and proportion of quantifiable PSMs for data acquired with conventional MS^2, conventional MS^3, and SPS-MS^3 with varying numbers of precursors. SPS-MS^3 with 10 precursors restores ion signal to levels comparable to MS^2, while improving the specificity and therefore accuracy of TMT quantification.

A

Fraction Number	Iodixanol Concentration (%)		
	**Experiment 1**	**Experiment 2**	**Experiment 3**
1	3.3	2.4	3.3
2	4.6	3.3	5.8
3	5.5	4.9	7.3
4	6.4	5.8	8.2
5	7.6	6.7	9.4
6	8.5	7.9	10.4
7	9.4	9.1	11.3
8	10.4	10.0	12.5
9	11.6	11.0	13.7
10	12.8	11.9	14.9
11	13.7	13.1	16.2
12	14.6	14.0	17.4
13	16.2	15.2	18.9
14	17.4	16.5	20.1
15	18.9	18.0	21.6
16	20.1	19.4	24.1
17	22.9	20.9	26.5
18	26.8	23.2	31.4
19	34.5	26.2	36.3
20	37.2	31.9	40.0
Cytosol*	0.0	0.0	0.0
Chromatin**	-	-	-

*****Not collected from density gradient. Cytosol enriched fractions were collected from the supernatant of the crude membrane preparation step, as described in the Methods section.
******Not collected from density gradient. Chromatin enriched fractions were prepared using a parallel enrichment strategy based on detergent permeabilization, as described in the Methods section.

B

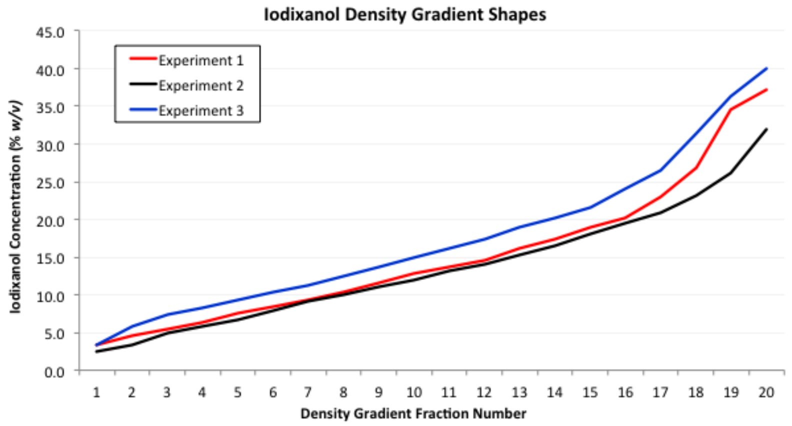

Supplementary Table 2 | Density gradient measurements (A) and plotted profiles (B) for three independent biological replicates.

TMT 10-plex Label	Density Gradient Fraction Number(s)			Subcellular Fraction Density (% *w/v* Iodixanol)		
	Experiment 1	Experiment 2	Experiment 3	Experiment 1	Experiment 2	Experiment 3
126	Cytosol	Cytosol	Cytosol	0.0	0.0	0.0
127N	1 to 6 (pooled)	1 to 6 (pooled)	1 to 4 (pooled)	6.0	5.2	6.2
127C	8 to 9 (pooled)	7 to 9 (pooled)	5 to 6 (pooled)	11.0	10.0	9.9
128N	10 to 11 (pooled)	10 to 11 (pooled)	8	13.3	12.5	12.5
128C	12	12	10	14.6	14.0	14.9
129N	14	14 to 15 (pooled)	12	17.4	17.3	17.4
129C	16	17	14	20.1	20.9	20.1
130N	18	18 to 19 (pooled)	17	26.8	24.7	26.5
130C	Chromatin	Chromatin	Chromatin	-	-	-
131	19	20	18	34.5	31.9	31.4

Supplementary Table 3 | Density gradient fractions selected for TMT 10-plex labeling in the three hyperLOPIT experiments.

Organelle	Number of proteins
ER/GA	76
Mitochondria	261
Plasma Membrane	50
Proteasome	34
Ribosome 40S	26
Ribosome 60S	43
unknown	3371

Supplementary Table 4 | Initial set of organelle marker proteins.

Organelle	Number of proteins
Phenotype 1	122
Phenotype 2	140
Phenotype 3	64
Phenotype 4	30
Phenotype 5	213
Phenotype 6	25
Phenotype 7	30
Phenotype 8	24
Phenotype 9	30
Phenotype 10	20
ER/GA	289
Mitochondria	449
Plasma Membrane	220
Proteasome	63
Ribosome 40S	63
Ribosome 60S	58
unknown	2021

Supplementary Table 5 | Assignments to novel phenotypes by the *phenoDisco* algorithm.

Organelle	Number of proteins
Actin cytoskeleton	13
Cytosol	43
Endosome	12
ER/GA	107
Lysosome	33
Mitochondria	383
Nucleus - Chromatin	64
Nucleus - Nucleolus	85
Peroxisome	17
Plasma Membrane	51
Proteasome	34
Ribosome 40S	27
Ribosome 60S	43
Unknown	2949

Supplementary Table 6 | Final augmented markers for SVM training.

Supplementary Note 1: Machine Learning Results

The first step of the classification process is to obtain a set of well-characterised organelle residents, termed protein 'markers'. These markers, once defined, can be used as input labelled data to train a machine learning classifier to assign proteins of unknown localisation to one of the localisations covered in the protein marker set. It is however laborious and extremely difficult to manually define reliable markers that cover the full sub-cellular diversity in the data, and furthermore to obtain markers that represent the true structure of any sub-cellular clusters determined, which is essential for sound analysis. As such, an initial round of phenotype discovery was conducted using the *phenoDisco* algorithm [13], in the *pRoloc* package [14].

Phenotype discovery

The *phenoDisco* algorithm uses iterative cluster merging combined with Gaussian Mixture Modelling and outlier detection, and with a minimal initial set of markers and unlabelled data can be used to effectively detect new putative clusters, beyond those that are initially manually described.

Ten new phenotype clusters were detected in the dataset (Supplementary Figure 15 and Supplementary Table 5). Each cluster was carefully validated by querying the UniProt database [15], the Gene Ontology [16] and the literature, as per the original pre-defined input markers, to assess biological relevance (Supplementary Table 6). Clusters that contained residents of small organellar structures such as the lysosome (phenotype 3) endosome (phenotype 4), and peroxisome (phenotype 7), were detected, thus confirming their independent data specific structure. Similarly, two very distinct nuclear clusters were confirmed, that were enriched in chromatin (phenotype 1) and nucleolus and other non-chromatin (phenotype 2) localised proteins. Further clusters contained actin cytoskeletal localised proteins (phenotype 9), ER localised proteins (phenotype 8) and a large cluster of cytosolic proteins (phenotype 5). We also see an interesting cluster that contains a small number of p-body proteins (phenotype 10) and a cluster of proteins that have mixed nuclear/cytoplasmic distributions (phenotype 6), of which many are known to shuttle between the nucleus and cytoplasm (see supplementary data 1 for *phenoDisco* output). Following examination of the phenotype clusters, further mining was conducted and well-known residents, as defined by UniProt and the literature, of the validated organelles were extracted and added to the list of protein markers to be used in a round of supervised machine learning classification. Markers for the lysosome, endosome, peroxisome, actin cytoskeleton, chromatin, nucleolus (non-chromatin) and cytosol were extracted from the discovery analysis to be added to the list of marker proteins. Proteins from phenotype 8, which are ER localised, were added to the existing set of ER markers, thus extending the number of markers for this organelle. Markers from phenotype 10 and phenotype 6 were left out of the final set of markers, as they were not highly enriched for one specific phenotype, and additionally the number of markers in these clusters was too small for use in classification (a minimum of 6 markers per subcellular class is required in supervised machine learning analysis for parameter optimisation as discussed in the proceeding section).

Increasing organellar resolution

Prior to novelty detection and supervised machine learning classification, to increase the organelle resolution, replicates 1 and 2 were combined using simple data fusion [17] in which quantitative TMT reporter ion ratios (10 per protein per experiment) were concatenated across the rows of proteins common in the two datasets. This combined dataset results in 20 quantitative data columns per protein and a total of 5032 proteins. Experiment 3 was not included as little additional resolution was obtained by further data fusion.

Comparison of MS^2 and SPS-MS^3 cluster resolution

Comparison of MS^2 and SPS-MS^3 protein-level cluster resolution and the repercussion for organelle proteomics has been investigated graphically as illustrated in Supporting Figures 17 and 18. The MS^2 and SPS-MS^3 (first replicate only) experiments contained 7116 and 5491 proteins respectively. Despite the higher number of proteins and peptide spectrum matches (PSMs) per proteins in MS^2, we demonstrate the negative impact of lack of accurate quantification on the sub-cellular resolution for proteins quantified by a limited number of PSMs. The histograms and density plots in Supplementary Figure 17, illustrate the higher number of proteins and PSM per protein in MS^2. Supplementary Figure 18 shows the SPS-MS^3 (top) and MS^2 (bottom) densities on the PCA plot for a set of PSM thresholds: from proteins with at least 20 PSMs per protein (left) to only a single PSM (right). Dense regions on the PCA plot are represented by darker shades on the figures. When considering proteins with a high number of PSMs (left), organelle clusters are clearly visible as darker groups. Filtering out proteins quantified by a high number of PMSs down to single PSM hits (right), the resolution of the sub-cellular clusters disappear already using a 5 PSM threshold in the MS^2 data; the density of point concentrates in the middle of the PCA figure, a pattern that characteristic of noisy, non-specific protein profiles. For SPS-MS^3 data, cluster resolution (organellar cluster densities and their separation) remains visible even for single PSM features.

Supplementary References

1 Bausch-Fluck, D. *et al.* A Mass Spectrometric-Derived Cell Surface Protein Atlas. *PLoS ONE* **10**, e0121314, doi:10.1371/journal.pone.0121314 (2015).

2 Braun, K. A. & Breeden, L. L. Nascent Transcription of MCM2-7 Is Important for Nuclear Localization of the Minichromosome Maintenance Complex in G(1). *Molecular Biology of the Cell* **18**, 1447-1456, doi:10.1091/mbc.E06-09-0792 (2007).

3 Betschinger, J. *et al.* Exit from Pluripotency Is Gated by Intracellular Redistribution of the bHLH Transcription Factor Tfe3. *Cell* **153**, 335-347 (2013).

4 Bouayad, D. *et al.* Nuclear-to-cytoplasmic Relocalization of the Proliferating Cell Nuclear Antigen (PCNA) during Differentiation Involves a Chromosome Region Maintenance 1 (CRM1)-dependent Export and Is a Prerequisite for PCNA Antiapoptotic Activity in Mature Neutrophils. *The Journal of Biological Chemistry* **287**, 33812-33825, doi:10.1074/jbc.M112.367839 (2012).

5 Krajewski, S. *et al.* Immunohistochemical analysis of Mcl-1 and Bcl-2 proteins in normal and neoplastic lymph nodes. *The American Journal of Pathology* **145**, 515-525 (1994).

6 Faou, P. & Hoogenraad, N. J. Tom34: A cytosolic cochaperone of the Hsp90/Hsp70 protein complex involved in mitochondrial protein import. *Biochimica et Biophysica Acta (BBA) - Molecular Cell Research* **1823**, 348-357, doi:http://dx.doi.org/10.1016/j.bbamcr.2011.12.001 (2012).

7 Gegonne, A. *et al.* TFIID component TAF7 functionally interacts with both TFIIH and P-TEFb. *Proceedings of the National Academy of Sciences of the United States of America* **105**, 5367-5372, doi:10.1073/pnas.0801637105 (2008).

8 Das, A. *et al.* RalA promotes a direct exocyst–Par6 interaction to regulate polarity in neuronal development. *Journal of Cell Science* **127**, 686-699, doi:10.1242/jcs.145037 (2014).

9 Havugimana, P. C. *et al.* A Census of Human Soluble Protein Complexes. *Cell* **150**, 1068-1081, doi:10.1016/j.cell.2012.08.011 (2012).

10 van den Berg, D. L. C. *et al.* An Oct4-Centered Protein Interaction Network in Embryonic Stem Cells. *Cell Stem Cell* **6**, 369-381, doi:10.1016/j.stem.2010.02.014 (2010).

11 Gagliardi, A. *et al.* A direct physical interaction between Nanog and Sox2 regulates embryonic stem cell self-renewal. *The EMBO Journal* **32**, 2231-2247, doi:10.1038/emboj.2013.161 (2013).

12 Gao, Z. *et al.* Determination of Protein Interactome of Transcription Factor Sox2 in Embryonic Stem Cells Engineered for Inducible Expression of Four Reprogramming Factors. *The Journal of Biological Chemistry* **287**, 11384-11397, doi:10.1074/jbc.M111.320143 (2012).

13 Breckels, L. M. *et al.* The effect of organelle discovery upon sub-cellular protein localisation. *Journal of Proteomics* **88**, 129-140 (2013).

14 Gatto, L., Breckels, L. M., Wieczorek, S., Burger, T. & Lilley, K. S. Mass-spectrometry-based spatial proteomics data analysis using pRoloc and pRolocdata. *Bioinformatics*, doi:10.1093/bioinformatics/btu013 (2014).

15 The UniProt, C. Reorganizing the protein space at the Universal Protein Resource (UniProt). *Nucleic Acids Research* **40**, D71-D75, doi:10.1093/nar/gkr981 (2012).

16 The Reference Genome Group of the Gene Ontology, C. The Gene Ontology's Reference Genome Project: A Unified Framework for Functional Annotation across Species. *PLoS Computational Biology* **5**, e1000431, doi:10.1371/journal.pcbi.1000431 (2009).

17 Trotter, M. W. B., Sadowski, P. G., Dunkley, T. P. J., Groen, A. J. & Lilley, K. S. Improved sub-cellular resolution via simultaneous analysis of organelle proteomics data across varied experimental conditions. *Proteomics* **10**, 4213-4219 (2010).
